# Genetic architecture of gene expression underlying variation in host response to porcine reproductive and respiratory syndrome virus infection

**DOI:** 10.1038/srep46203

**Published:** 2017-04-10

**Authors:** Arun Kommadath, Hua Bao, Igseo Choi, James M. Reecy, James E. Koltes, Elyn Fritz-Waters, Chris J. Eisley, Jason R. Grant, Robert R. R. Rowland, Christopher K. Tuggle, Jack C. M. Dekkers, Joan K. Lunney, Le Luo Guan, Paul Stothard, Graham S. Plastow

**Affiliations:** 1 Department of Agricultural, Food and Nutritional Science, University of Alberta, Edmonton T6G 2P5, AB, Canada; 2 Department of Research and Development, Geneseeq Technology Inc., Toronto M5G 1L7, ON, Canada; 3 USDA-ARS, BARC, APDL, Building1040, Beltsville 20705, MD, USA; 4 Department of Animal Science, Iowa State University, 2255 Kildee Hall, Ames 50011, IA, USA; 5 Department of Animal Science, University of Arkansas, AFLS B106D, Fayetteville, AR, 72703, USA; 6 Department of Statistics, Iowa State University, 1121 Snedecor Hall, Ames, IA 50011, USA; 7 College of Veterinary Medicine, Kansas State University, K-231 Mosier Hall, Manhattan 66506, KS, USA

## Abstract

It has been shown that inter-individual variation in host response to porcine reproductive and respiratory syndrome (PRRS) has a heritable component, yet little is known about the underlying genetic architecture of gene expression in response to PRRS virus (PRRSV) infection. Here, we integrated genome-wide genotype, gene expression, viremia level, and weight gain data to identify genetic polymorphisms that are associated with variation in inter-individual gene expression and response to PRRSV infection in pigs. RNA-seq analysis of peripheral blood samples collected just prior to experimental challenge (day 0) and at 4, 7, 11 and 14 days post infection from 44 pigs revealed 6,430 differentially expressed genes at one or more time points post infection compared to the day 0 baseline. We mapped genetic polymorphisms that were associated with inter-individual differences in expression at each day and found evidence of *cis-*acting expression quantitative trait loci (*cis-*eQTL) for 869 expressed genes (qval < 0.05). Associations between *cis*-eQTL markers and host response phenotypes using 383 pigs suggest that host genotype-dependent differences in expression of *GBP5, GBP6, CCHCR1 and CMPK2* affect viremia levels or weight gain in response to PRRSV infection.

Porcine reproductive and respiratory syndrome (PRRS) virus, or PRRSV, is a positive-strand RNA virus that belongs to the Arteriviridae family^1^. PRRSV causes decreased reproductive performance and respiratory problems in pigs, which result in significant economic losses in the swine industry^2^,^3^. Individual pigs vary in susceptibility to PRRSV infection and several single nucleotide polymorphism (SNP) markers were found to be associated with viremia levels (VL) and weight gain (WG) by genome-wide association studies (GWAS)^4^,^5^. For example, a quantitative trait locus (QTL) in high linkage disequilibrium (LD) with the SNP WUR10000125 (WUR) was identified on *Sus scrofa* chromosome 4 (SSC4) that explained a considerable amount of the total genetic variance for VL (13.2%) and WG (9.1%) of weaned piglets following experimental infection^4^. Nine additional regions were reported to explain a further 5.2% and 8.5% of the genetic variance for VL and WG, respectively^4^. A recent study of gene expression in this QTL region identified a putative quantitative trait nucleotide in the guanylate binding protein 5 (*GBP5*) gene, that creates alternative splicing of GBP5 RNA, which decreased wild type RNA levels expressed from the unfavorable allele^6^. Studies of *GBP5* knockout mice indicated that *GBP5* functions in host defense, inflammasome assembly, and inflammatory responses to pathogenic bacteria^7^ and recently another study reported that *GBP5* potently restricts HIV-1 and other retroviruses^8^. Thus the predicted loss of wild type GBP5 expression from the unfavorable allele is consistent with the poor outcome of homozygous individuals following PRRSV infection. However, candidate causal genes in the other nine regions are still unknown.

Variation in gene expression among individuals has a strong genetic component^9^, and specific polymorphic loci affecting gene expression, known as expression quantitative trait loci (eQTL), have been reported^10^. Responses to pathogen invasion and immunity to infection require coordinated regulation of gene expression^11^. Recent studies indicate that variation in expression levels of genes involved in immune responses are associated with regulatory variants^12^. For example, Barreiro *et* *al*.^13^ infected monocyte-derived dendritic cells from 65 human individuals with *Mycobacterium tuberculosis* and identified several polymorphisms associated with variation in cytokine expression, including *CXCR1, IL1Ra* and *IL15*. Further, they integrated eQTL data with results from a previous GWAS for pulmonary tuberculosis and identified a promising candidate gene, *DUSP14*, that may underlie susceptibility to *Mycobacterium tuberculosis* infection^13^. There is increasing evidence to indicate that SNPs associated with complex traits are likely to be eQTLs^14^,^15^. In this study, we aimed to identify genes and mechanisms that affect the susceptibility to PRRSV infection through the integration of eQTL and GWAS analyses. Our results lend further support to the important role of *GBP5* in host response to PRRSV infection and also identified additional candidate genes within the top GWAS regions associated with VL and WG reported in earlier studies^4^,^5^,^6^.

## Results

### Temporal transcriptional response to PRRSV infection

To study gene expression dynamics during PRRSV infection, we used data from two independent virus challenge trials, which involved 44 pigs that were infected by PRRSV isolate NVSL97-7985. Detailed information on the experimental pigs is provided in Supplementary Tables S1A and S1B. Illumina paired-end sequences from 190 blood RNA samples collected at time points 0 (just prior to experimental infection), 4, 7, 11 and 14 days post infection (DPI) were retained. Approximately 84% of the 4.2 billion sequenced reads (an average of 22 million paired-end reads per sample) were mapped to the pig reference genome (Sscrofa10.2)^16^. Following sample and gene filtering steps, a set of 8863 genes was identified as expressed in porcine peripheral blood across the 190 samples. Using a generalized linear model, 6430 genes were declared differentially expressed (DE) in response to PRRSV infection for at least one DPI compared to the day 0 baseline (Benjamini-Hochberg corrected p-value < 0.05). The largest number of DE genes was observed at 4 DPI (4753 genes). Similar (or even larger) numbers of infection responsive or DE genes have been reported post infection in previous studies on PRRS^17^ and other infections^13^,^18^.

**Figure 1:**
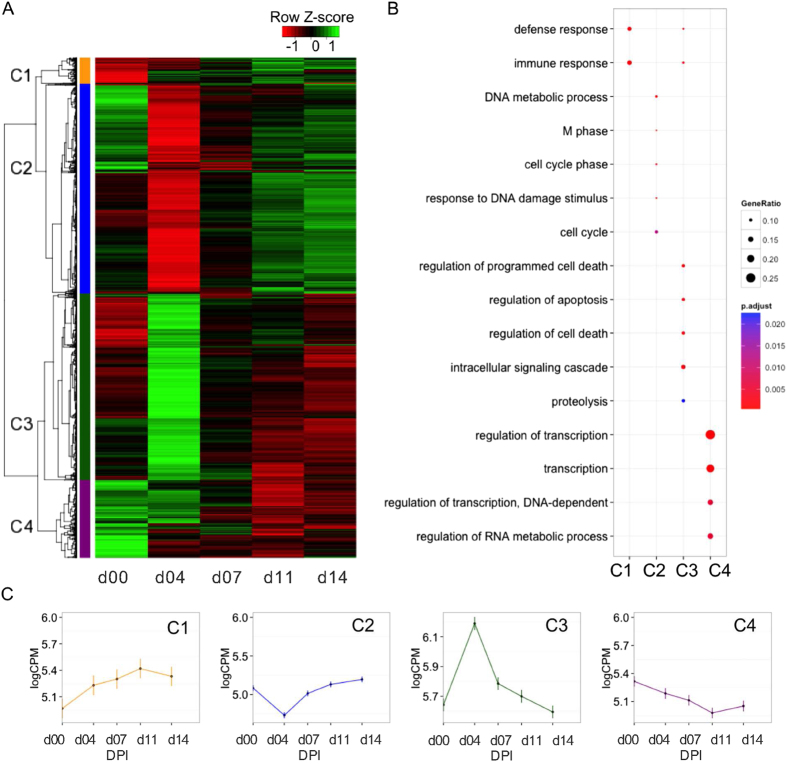
Differentially expressed genes in response to PRRSV infection. (**A**) Heatmap depicting average expression per day (log2 CPM) of 6430 genes identified as DE in response to PRRSV infection for at least one day compared to day 0 expression levels (FDR < 0.05). The 4 major clusters identified are labeled as C1 to C4. (**B**) Enriched GO biological process terms within each of the 4 clusters. (**C**) Representative expression pattern for each of the 4 clusters across days with standard error bars (based on average expression per day (log2 CPM) of all genes within that cluster).

Hierarchical clustering of these DE genes by their log-average abundance per day (derived from log-average abundance at day 0 and ratios of log-abundance at other DPI relative to day 0) revealed four broad clusters with distinct expression profiles (Fig. 1A and C). The biological functions that represented each cluster were determined by gene ontology (GO) enrichment analysis, taking the set of all expressed genes as the reference set. The expression level of cluster 1 (C1) genes, which were enriched for the GO term immune response, increased following infection; whereas, the expression level of cluster 4 (C4) genes, which were enriched for the GO term regulation of transcription, showed the opposite trend (Fig. 1B and C). The expression level of cluster 2 (C2) genes, which were enriched for GO terms DNA metabolic process and cell cycle, showed a sharp decrease at day 4; whereas, the expression level of cluster 3 (C3) genes, which were enriched for the GO terms regulation of apoptosis and intracellular signaling cascades, showed a sharp increase at day 4 (Fig. 1B and C). The complete list of enriched GO terms per cluster is provided in Supplementary Table S2.

### Mapping *cis*-eQTLs in response to PRRSV infection

In addition to the gene expression data described above, PorcineSNP60 BeadChip genotype data (see Methods) for the same set of 44 pigs were available as part of the previously reported GWAS^4^. The availability of these two datasets made it possible to perform expression quantitative trait loci (eQTL) analyses to help identify genetic markers associated with gene expression variation during PRRSV infection.

In this study, we tested genotype-gene expression associations separately for each DPI and for each of the 8863 expressed genes. Given the small sample size in each analysis (n = 44) and hence the relatively low statistical power, we did not test for *trans*-eQTL and only focused on *cis*-eQTL between each autosomal gene and SNPs in its *cis*-candidate region (from 1 Mb upstream of the transcription start site to 1 Mb downstream of the transcription end site). Since the 44 pigs were selected from two different genetic backgrounds (16 from trial PHGC3 and 28 from trial PHGC5), we performed a principal component analysis (PCA) on the genotype data to detect population structure. We observed that the first principal component (PC) clearly separated pigs from the two trials and the second PC separated pigs from PHGC5 into three subgroups (Supplementary Fig. S1). We also performed PCA on the regularized log (rlog; see Methods) transformed expression matrix from all 190 samples. The first three PCs explained ~70% of the variance in the expression data (Supplementary Fig. S2). Here too, the first PC separated the pigs by trial, whereas the third PC clearly separated the 4 DPI samples from the others. Probabilistic Estimation of Expression Residuals (PEER) software was used to identify hidden confounding factors based on the rlog transformed expression matrix and known covariates (see Methods) and to generate residual expression levels. We performed an eQTL analysis (see Methods), testing associations between SNP genotypes and residual expression levels, and found 579, 902, 1269, 566 and 1174 unique SNPs showing significant *cis*-eQTL signals (qval < 0.05) at 0, 4, 7, 11 and 14 DPI respectively. The *cis*-eQTL SNPs for the five time points were linked to 198, 278, 439, 205 and 392 unique *cis*-genes, respectively, i.e. genes whose expression levels were associated with variation at a particular SNP. In total, we identified 2523 unique SNPs and 869 unique genes with evidence of *cis*-eQTL signals at one or more DPI.

### Identifying *cis*-eQTL SNPs and *cis*-genes associated with VL and WG

Next, we sought to identify *cis*-eQTL that may affect VL or WG by determining the overlap between locations of the 2523 unique *cis*-eQTL SNPs identified here and the top genomic regions of 1 Mb each that were reported to be associated with the same traits in a previous GWAS^4^ (Supplementary Table S3; see Methods). A total of 12 and 23 of our *cis*-eQTL SNPs (Supplementary Table S4) overlapped with the top 8 autosomal regions that were reported in that GWAS for VL and WG, respectively (note that the X chromosome was excluded from our study due to insufficient power to perform eQTL analysis separately for males and females).

Not all of the 12 and 23 *cis*-eQTL SNPs that overlapped with the GWAS regions are expected to be associated with VL or WG. Thus, to refine this set of SNPs to obtain trait-associated candidate *cis*-eQTL SNPs, we tested their associations (see Methods) with VL and WG separately at each DPI (4, 7, 11 and 14 for VL; 7, 14, 28, 35 and 42 for WG) using data from 383 animals (from trial PHGC3 and PHGC5) for which both genotype and phenotype data were available. Based on this, we identified a refined set of 11 and 12 candidate *cis*-eQTL SNPs (with an intersect of 7 SNPs) that were associated with VL and WG respectively at one or more DPI (Supplementary Table S4). Further investigation of the *cis*-genes linked to these trait-associated candidate *cis*-eQTL SNPs, identified 5 candidate *cis-*genes (*CD1D, FCER1A, FCRL6, GBP5, GBP6*) within 2 GWAS regions for VL and 5 candidate *cis-*genes (*CCHCR1, CMPK2, GBP5, ICE2, PPT2*) within 4 GWAS regions for WG (Table 1). The set of 7 *cis*-eQTL SNPs that were found associated with both traits fell within the *cis*-regulatory region of *GBP5* and the GWAS region at 139–140 Mb on SSC4.

### Candidate genes underlying variation in host response to PRRSV infection

**Figure 2:**
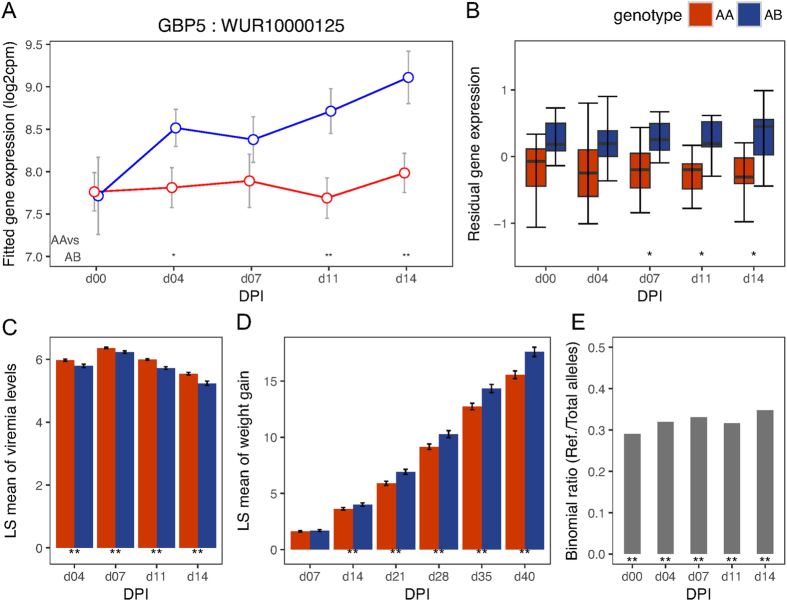
Temporal expression patterns, eQTL, phenotype associations and ASE for *GBP5.* (**A**) Points in the plot represent the mean of the log2 transformed gene expression (from the day effect model) and error bars represent the range at 95% confidence interval at different DPI for the genotypes based on its *cis-*eQTL (WUR10000125). Symbols at the base of the plot represent the p-value of the Welch two sample t-test performed to test the alternative hypothesis that the true difference in means is less than 0 between AA and AB genotypes (**<0.01; *<=0.05; ^**+**^<0.1). (**B**) Box plot showing differences in residual expression levels of *GBP5* between AA (n = 26) and AB (n = 17) genotypes of its *cis*-eQTL by DPI. (**C**) Bar plot showing differences in least square means (adjusting for gender and population structure) for VL (log_10_(templates/reaction)) between genotypes (AA: n = 247, AB: n = 124) at marker WUR10000125 across days. (**D**) Bar plot showing differences in least square means for WG (kg) between genotypes (AA: n = 247, AB: n = 124) at marker WUR10000125 across days. (**E**) Bar plot showing the allelic imbalance at heterozygous exonic SNPs of *GBP5* that are in phase with the reference allele of its *cis*-eQTL across days. The susceptible allele A corresponds to the reference allele. Symbols at the base of plots B–E indicate the level of significance of the association tested at that DPI (**<0.01; *<=0.05). Information from BB genotype animals is not shown in any of the plots due to low representation in the test population (Only 1 of 44 in the gene expression and eQTL associations and 12 of 383 in the trait associations).

**Figure 3:**
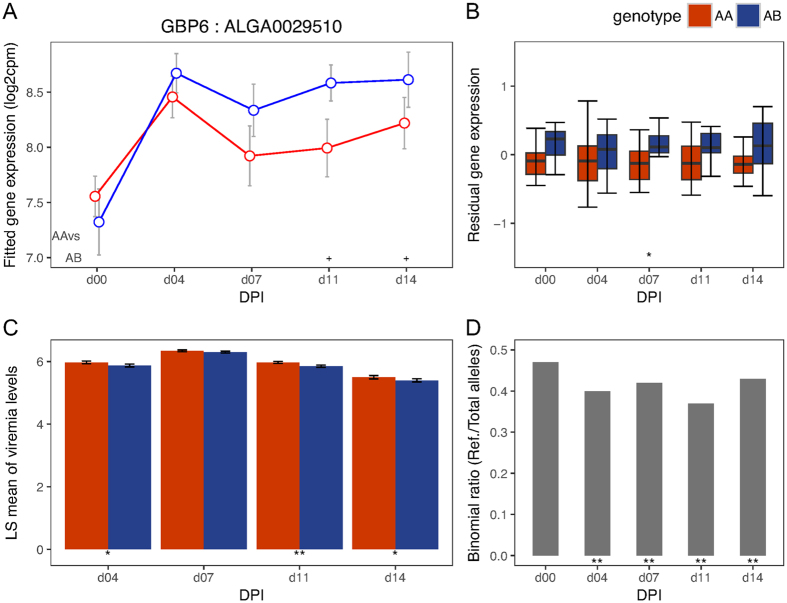
Temporal expression patterns, eQTL, phenotype associations and ASE for *GBP6.* (**A**) Points in the plot represent the mean of the log2 transformed gene expression (from the day effect model) and error bars represent the range at 95% confidence interval at different DPI for the genotypes based on its *cis-*eQTL (ALGA0029510). Symbols at the base of the plot represent the p-value of the Welch two sample t-test performed to test the alternative hypothesis that the true difference in means is less than 0 between AA and AB genotypes (**<0.01; *<=0.05; ^**+**^<0.1). (**B**) Box plot showing differences in residual expression levels of *GBP6* between AA (n = 22) and AB (n = 21) genotypes of its *cis*-eQTL by DPI. (**C**) Bar plot showing differences in least square mean (adjusting for gender and population structure) of VL (log_10_(templates/reaction)) among different genotypes (AA: n = 175, AB: n = 184) of marker ALGA0029510 across days. (**D**) Bar plot showing the DPI wise allelic imbalance at the heterozygous exonic SNPs of *GBP6* in phase with the reference allele of its *cis*-eQTL. The susceptibility allele A corresponds to the reference allele. Symbols at the base of plots B–D indicate the level of significance of the association tested at that DPI (**<0.01; *<=0.05). Information from BB genotype animals is not shown in any of the plots due to low representation in the test population (Only 1 of 44 in the gene expression and eQTL associations and 24 of 383 in the trait associations).

**Figure 4:**
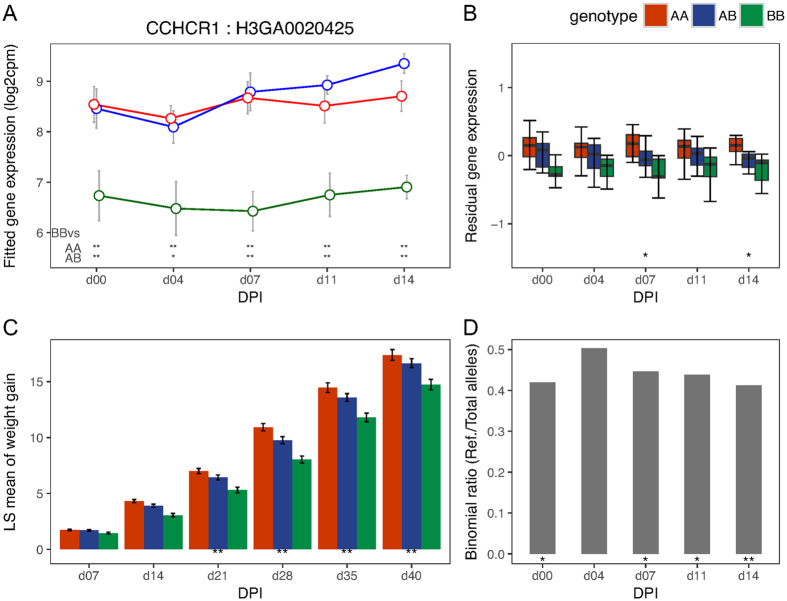
Temporal expression patterns, eQTL, phenotype associations and ASE for *CCHCR1.* (**A**) Points in the plot represent the mean of the log2 transformed gene expression (from the day effect model) and error bars represent the range at 95% confidence interval at different DPI for the genotypes based on its *cis-*eQTL (H3GA0020425). Symbols at the base of the plot represent the p-value of the Welch two sample t-test performed to test the alternative hypothesis that the true difference in means is less than 0 between BB and AA or AB genotypes (**<0.01; *<=0.05; ^**+**^<0.1). (**B**) Box plot showing differences in residual expression levels of *CCHCR1* between AA (n = 18), AB (n = 15) and BB (n = 11) genotypes of its *cis*-eQTL across days. (**C**) Bar plot showing differences in least square mean of WG (kg) among different genotypes (AA: n = 100, AB: n = 169, BB: n = 114) of marker H3GA0020425 across days. (**D**) Bar plot showing the DPI wise allelic imbalance at the heterozygous exonic SNPs of *CCHCR1* in phase with the reference allele of its *cis*-eQTL. The susceptibility allele B corresponds to the reference allele. Symbols at the base of plots B–D indicate the level of significance of the association tested at that DPI (**<0.01; *<=0.05).

**Figure 5:**
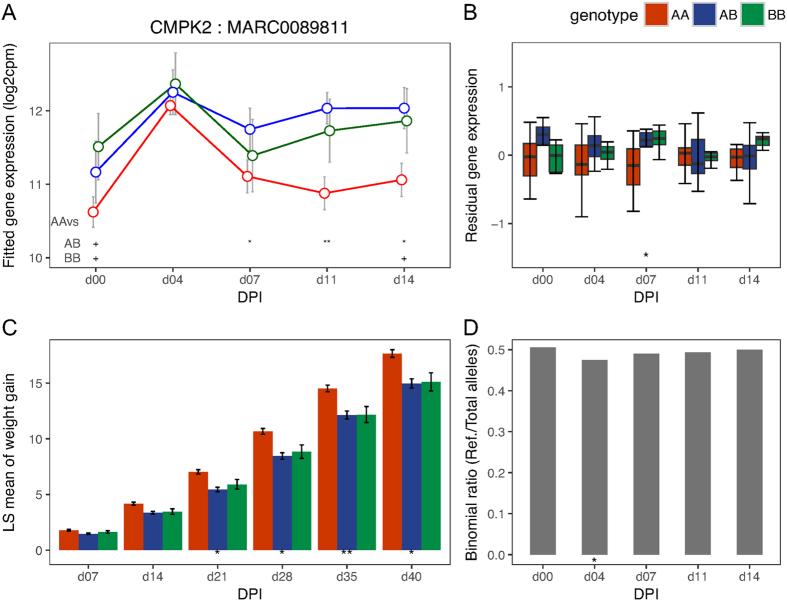
Temporal expression patterns, eQTL, phenotype associations and ASE for *CMPK2.* (**A**) Points in the plot represent the mean of the log2 transformed gene expression (from the day effect model) and error bars represent the range at 95% confidence interval at different DPI for the genotypes based on its *cis-*eQTL (MARC0089811). Symbols at the base of the plot represent the p-value of the Welch two sample t-test performed to test the alternative hypothesis that the true difference in means is less than 0 between AA and AB or BB genotypes (**<0.01; *<=0.05; ^**+**^<0.1). (**B**) Box plot showing differences in residual expression levels of *CMPK2* between AA (n = 18), AB (n = 15) and BB (n = 11) genotypes of its *cis*-eQTL across days. (**C**) Bar plot showing differences in least square mean of WG (kg) among different genotypes (AA: n = 185, AB: n = 162, BB: n = 36) of marker MARC0089811 across days. (**D**) Bar plot showing the DPI wise allelic imbalance at the heterozygous exonic SNPs of *CMPK2* in phase with the reference allele of its *cis*-eQTL. The susceptibility allele B corresponds to the non-reference allele. Symbols at the base of plots (**B**–**D**) indicate the level of significance of the association tested at that DPI (**<0.01; *<=0.05).

To validate the *cis*-eQTL SNPs identified above, we tested for evidence of allele-specific expression (ASE) at their *cis-*genes after first correcting for bias associated with mapping reads that contain the non-reference allele to the reference genome (see Methods). We found strong evidence of ASE (p < 0.01) for all *cis*-eQTL SNPs for *GBP5* at almost all DPI (including day 0), with the WUR SNP among the most significant (p < 1e-06) *cis*-eQTL SNPs (Fig. 2). The other gene in the same GWAS region, *GBP6*, showed strong evidence of ASE (p < 0.01) at all time-points post infection (Fig. 3). The *CD1D* gene did not show evidence of ASE at any DPI. The gene *CCHCR1* (Fig. 4) showed evidence of ASE across multiple DPI (p = 0.03 at day 0; p = 0.04 at 7 DPI; p = 0.05 at 11 DPI; p = 0.005 at 14 DPI), while *CMPK2* (Fig. 5) showed evidence of ASE at 4 DPI alone (p = 0.05). The other candidate genes (*FCER1A, FCRL6, ICE2, PPT2*) could not be tested for ASE due to inadequate read depth at their exonic SNPs. Details of the ASE test results are provided in Supplementary Table S5. We thus narrowed the list of candidate genes that underlie variation in host response to PRRSV infection within the top GWAS regions for VL and WG traits down to those validated with ASE, i.e., *GBP5, GBP6, CCHCR1* and *CMPK2*. The DE, eQTL, phenotype associations, and ASE for these final candidate genes for VL and WG are shown in Figs 2, 3, 4, 5, with detailed statistics provided in Supplementary Table S6. For *GBP5, GBP6* and *CCHCR1*, the susceptibility allele (allele associated with higher VL and/or lower WG) was associated with lower expression at their respective *cis*-eQTLs and genes, whereas for *CMPK2*, the susceptibility allele showed the opposite trend in expression. In general, for VL, differences among genotypes were observed at the same and/or subsequent DPIs as when the significant eQTL was observed whereas for WG, the differences were not immediately apparent as can be expected.

## Discussion

A large proportion of genetic variance for VL and WG following experimental PRRSV infection of weaner pigs was explained by several markers that were in strong LD in the Chr4:139 Mb region^4^,^5^. A recent study^6^ showed that pigs with the susceptible genotype (Allele A of WUR10000125, associated with increased VL) expressed *GBP5* at a lower level than the resistant genotype and primarily produced an alternate splice form that was predicted to produce a truncated protein. That study focused on the WUR region and tested differential expression and splicing using the 16 animals from trial PHGC3 and validated the results using the 28 animals from trial PHGC5. The host-genotype dependent expression differences for *GBP5* reported in that study were confirmed here, where we combined these two data sources and integrated GWAS results with eQTL and ASE analyses. Further, we hypothesize that the eQTL for *GBP5* provides control at the post-transcriptional level. Allele A at SNP WUR10000125 is in extremely high LD with a premature stop codon (111 bp upstream of the last exon-exon junction) in the coding region of *GBP5*^6^. If a transcript harbors a premature termination codon whose location is > 50 bp upstream of the final exon-exon junction, it is highly likely to be degraded by the nonsense-mediated decay (NMD) pathway^19^. Thus, the *cis*-eQTL for *GBP5* (lower expression associated with the A allele) can potentially be explained by NMD of the RNA transcript that is preferentially expressed by the A allele.

This study also aimed to identify additional candidate genes that underlie variation in phenotypes (VL and WG) through our integrated eQTL analyses approach. One such candidate gene for VL is *GBP6*. Although we consider *GBP5* as a better candidate gene for PRRS response than *GBP6* in the chr4:139 Mb region, the fact that *GBP6* was upregulated after infection and showed evidence of ASE at all time-points post infection indicates that its *cis*-eQTL may respond to viral infection. These and other members of the GBP family are interferon-inducible GTPases that are known to be involved in immunity against intracellular bacteria, protozoa and viruses^20^,^21^,^22^. Other researchers, analysing limited numbers of type 2 PRRSV infected pigs, identified a SNP in *GBP1* but not WUR to be associated with increased WG and lower VL^23^.

The other candidate genes for VL, *FCRL6* and *FCER1A*, could not be tested for ASE due to lack of heterozygous exonic SNPs called from our RNA-Seq data for those genes. While *FCRL6* was down regulated at all DPI compared to 0 DPI, the expression of *FCER1A* did not change initially but was slightly upregulated at later DPI. Both *FCRL6* and *FCER1A* encode Fc receptors that are involved in immunity and our data showed a higher expression of the susceptibility allele at their *cis*-eQTLs, which might indicate their involvement in some aspect of signalling that is induced by the virus. A previous GWAS in humans identified *FCER1A* as a susceptibility locus that influences total serum IgE^24^. Moreover, *FCRL6* and *FCER1A* are also likely to act in a negative regulatory role to limit the pro-inflammatory environment that is established during anti-viral immune response, as has been shown for several members of the family of Fc receptors^25^,^26^.

Among our candidate genes for WG, *CMPK2* has been reported to be associated with monocytic/macrophage terminal differentiation^27^ and *CCHCR1* might function in EGFR-STAT3 signaling and innate immunity^28^. The gene *CCHCR1* was significantly down-regulated at 4 DPI compared to day 0 and was expressed at a lower level at all DPI in individuals homozygous for the susceptibility allele. In contrast, the gene *CMPK2* was over two-fold up-regulated at 4 DPI compared to day 0 and was expressed at a higher level in individuals carrying the susceptibility allele, with the greatest differences observed at DPI at or following the DPI corresponding to its significant *cis*-eQTL. These observations might indicate that a favorable response for the host would be one where the infection responsive effects (suppression or induction) at these candidate genes are controlled. The other candidate genes for WG, *ICE2* and *PPT2*, could not be tested for ASE due to lack of heterozygous exonic SNPs called from our RNA-Seq data. Not much is known about the role of *ICE2* and *PPT2* in immunity but *PPT2* is annotated to pathways related to fatty acid metabolism, which are known to be induced in the host by many viruses^29^. Both *PPT2* and *CCHCR1* and their *cis*-eQTLs fall within the SLA/MHC complex of the pig genome^30^,^31^.

A recent GWAS^32^ used single-SNP and Bayes-B methods to jointly analyze data from 13 PHGC trials on piglet responses to infection with one of two PRRSV isolates (NVSL and KS06), including the PHGC3 and PHGC5 trials that were used here. When comparing results for the NVSL isolate from the Bayes-B method performed in that GWAS with those from Boddicker *et al*.^4^ (same isolate and similar model using Bayes-B method as Waide *et al*., but analysed data from only the first 8 PHGC trials), only the WUR region (chr4:139 Mb), the chr1:292 Mb region for VL and the chr3:138 Mb region for WG were reported in both studies. Three of the final four candidate genes we identified here fall within those common regions (*GBP5* and *GBP6* for VL within the WUR region; *CMPK2* for WG within the chr3:138 Mb region).

To conclude, we identified four strong candidate susceptibility genes *GBP5, GBP6, CCHCR1* and *CMPK2*, that were associated with higher viremia levels or lower weight gain in response to PRRSV infection and which also showed evidence of allele-specific expression. Although further studies are needed to define the causal mutations that regulate the expression of these candidate susceptibility genes, the current findings improve our understanding of disease control mechanisms and may aid in the development of new diagnostic tools for veterinarians or contribute to strategies for selection and breeding of pigs with improved resistance to PRRSV infections. In addition to these candidates, we found numerous genetic variants associated with inter-individual variation in the expression of several interferon signaling genes and genes with known anti-viral effects, including *OAS1, OAS2, USP18, MX1*, and several interleukin genes. These candidate *cis*-eQTLs may contribute to variation in immune response to PRRSV or even other pathogenic infections and may be associated with traits not tested here, thus warranting future research in that direction.

## Materials and Methods

### Animal information, sample collection and processing and phenotype recordings

The genetic backgrounds of pigs used in this study were described in a previous study^4^. Briefly, pigs in trial 3 (PHGC3) were F1 individuals from a Large White (LW) and Landrace (LR) cross^4^ and pigs in trial 5 (PHGC5) were a cross between Duroc sires and F1 LR/Yorkshire dams^4^ (~200 commercial crossbred piglets per trial). After a seven-day acclimation period, pigs were experimentally infected intramuscularly and intranasally with a 10^5^ tissue culture infectious dose50 of NVSL 97–7985 (a highly virulent PRRSV isolate) and followed for 42 days post infection. Blood samples for RNA isolation were collected at 0, 4, 7, 11, and 14 days post infection (DPI) using Tempus^TM^ Blood RNA Tubes (Life Technologies, Carlsbad, CA, USA). Serum samples from the same DPI were used to quantify viremia levels (VL) as log10 transformed viral template copies per amplification reaction using a semi-quantitative TaqMan PCR assay for PRRSV RNA. The pigs’ body weights were measured to quantify weight gain (WG) defined as the difference in weight (at day 7, 14, 28, 35 and 42) from weight on day 0. All pigs were genotyped using the Illumina PorcineSNP60 BeadChip. All animal experiments were approved by the Kansas State University Institutional Animal Care and Use Committee under registration number 3000 and were performed in accordance with approved guidelines and regulations.

Our study included 16 pigs from PHGC3 (used to analyse gene expression in WUR region in a previous study^6^) from 8 litters (one AA and one AB individual at the WUR SNP from each litter), along with 28 pigs (18 AA, 9 AB and 1 BB individuals at the WUR SNP) that were selected from the PHGC5 trial based on maximizing the number of animals with the AB genotype that had quality RNA samples for most/all days, while representing phenotypic data groups with contrasting viral responses (fast versus slow viral clearance). Total RNA of blood samples obtained from these pigs was isolated using the Tempus Spin RNA isolation Kit (Life Technologies, Carlsbad, CA, USA) and MagMax for Stabilized Blood Tubes RNA Isolation Kit for Tempus tubes (Life Technologies), respectively, according to the respective manufacturer’s instructions. RNA concentration was quantified using a ND-1000 spectrophotometer (NanoDrop Technologies, Wilmington, DE, USA) and RNA quality was assessed using either Agilent 2100 Bioanalyzer or 2200 TapeStation (Agilent Technologies, Inc., Santa Clara, CA, USA). The globin transcripts (HBA and HBB) were reduced using an RNase H based globin reduction method^33^ and RNA quality was assessed again.

### Library Preparation and RNA-sequencing

Poly (A)^+^ fractions from the globin depleted RNA samples (1.0 μg RNA each) were purified by oligo-dT purification beads (Illumina, Inc., San Diego, USA) and then used to construct cDNA libraries following the TruSeq RNA Sample Preparation Guide (Illumina, Inc., San Diego, USA). Sequencing was performed on the HiSeq 2000 System (Illumina, Inc.) using the TruSeq Paired-End (PE) 100 bp Kit (Illumina, Inc.). Real-time analysis and base calling were performed using the Control Software on the instrument. Initial processing of reads from the HiSeq machine used the Illumina CASAVA (v1.8) software.

### Differential expression analysis of RNA-seq

Sequence reads with base quality scores were produced by the Illumina sequencer. RNA-Seq reads flagged as low quality by the chastity filter in CASAVA 1.8 were removed. In addition, we removed reads with an average read quality score below 15 and reads in which over 5 of the last 10 bases had a PHRED quality score below 2. Sequence reads were aligned to pig reference genome sequence assembly Sscrofa10.2 using TopHat 2.0.12^34^ with default parameters, which included: enable use of GTF file, set minimum anchor length of 8, accept zero mismatches in the anchor region, allow intron length between 50 and 500,000, and allow up to 20 alignments to the reference for a given read. For annotation of genes, we used the GTF file for Sscrofa10.2 from Ensembl version 71. The number of reads uniquely mapped to each gene was determined using the htseq-count script of the HTSeq package (v0.5.3.p3)^35^. Reads that were assigned to more than one gene were not counted by htseq-count.

For further processing of the read counts, we used the Bioconductor (version 2.26.0)^36^ package edgeR (version 3.8.5)^37^ of the R (version 3.1.2) statistical programming language^38^. Read counts per gene were normalised to counts per million (CPM). Genes expressed at very low levels were removed by keeping only genes with a CPM greater than four in at least half the samples, resulting in an expression data set with 8863 genes. Trimmed mean of M-values (TMM) normalisation^39^ was applied to this dataset to account for compositional differences between the libraries. Genes that responded to PRRSV infection at any of the days after infection compared to the day 0 baseline were determined in edgeR using a generalized linear model that took the following factors into account: day, gender, trial (PHGC3 versus PHGC5), post globin depletion RIN score, and population structure (covariates for the first three PCAs based on the SNP genotypes of the 44 animals). Multiple test correction was conducted using the FDR calculation of Benjamini and Hochberg^40^ and the FDR cut-off for significance was set to 0.05. Genes that were responsive to PRRSV infection were clustered by hierarchical clustering using the average linkage method and correlation distance measures using the *hclust* function within stat and the *heatmap*.2 function within the gplot packages in R. Gene ontology (GO)^41^ biological process terms enriched in each cluster compared to a background set of all genes expressed in porcine blood (n = 8863) were determined using DAVID^42^ and the Bioconductor package clusterProfiler (version 2.2.3)^43^. Human orthologs of the corresponding porcine genes were used in the GO enrichment tests to take advantage of the more complete GO annotation available for human genes.

### Identification of *cis*-eQTLs and trait-QTLs

All pigs were genotyped using Illumina’s PorcineSNP60 BeadChip. We tested associations of SNP genotype with gene expression phenotypes (all expressed genes) separately for each DPI after applying the following filters on SNPs. First, we removed 17,065 of the total 62,163 SNPs in the genotype data that either had a minor allele frequency (MAF) below 12.5% or had a genotype call score below 0.15 in over 4 of the 44 samples. Of the remaining SNPs, 38907 were autosomal SNPs with known genomic positions. Due to the limited sample size and insufficient power to perform eQTL analysis separately for males and females, we tested only *cis*-associations for each gene based on autosomal SNPs in its *cis*-regulatory region (from 1 Mb upstream of the transcription start site to 1 Mb downstream of the transcription end site). Probabilistic Estimation of Expression Residuals (PEER)^44^ software (version 1.0) was used to identify hidden confounding factors in the normalised expression data by including known covariates (the same as used in the DE analysis) separately for each DPI. The expression dataset was variance-stabilized before PEER correction using the regularized log (rlog) transformation function in Bioconductor package DESeq2 (version 1.12.4)^45^. PEER then generated residual expression levels after subtracting known and estimated hidden factor contributions. We used MatrixEQTL (version 2.1.1)^46^ to conduct *cis*-eQTL mappings directly on residual expression levels, separately for each DPI, by linear regression of gene expression on genotype at *cis*-SNPs, one at a time. To account for LD among SNPs, we generated p-values from a null distribution by running the MatrixEQTL analysis 10,000 times, while permuting the sample labels of the expression matrix for each replicate. To control for FDR, q-values were generated from the null p-values using the qvalue package^47^ in R; a q-value < 0.05 was used as the criterion to select significant *cis*-eQTL SNPs.

A previous GWAS^4^ where the PorcineSNP60 genotypes from pigs belonging to the first 8 PHGC trials (including PHGC3 and PHGC5) were tested for associations with VL up to day 21 (area under the curve from day 0 to 21 DPI) and WG from day 0 to 42 DPI reported the top 10 most significant genomic regions associated with each trait (results summarized in Supplementary Table S3). We tested the association between *cis-*eQTL SNPs (MAF > 10%) that fall within the top 10 genomic regions reported in that GWAS for VL and WG separately at each DPI using data from 383 animals (188 from trial PHGC3 and 195 from trial PHGC5) for which both genotype and phenotype data were available (note that RNA-seq was performed on 44 animals selected from this set of 383 animals). This test was based on additive linear regression model, controlling for the effects of gender and population structure (covariates on the first three PCAs based on complete SNP genotype data of all 383 animals from PHGC3 and PHGC5). Since our association tests were limited to *cis*-eQTL SNPs within regions that were previously identified as associated with VL and WG by GWAS, we used a raw p-value cut-off of 0.01 (without multiple testing corrections) as our criteria for significance.

### Allele-specific expression (ASE) analysis

VarScan v2.3.6^48^ was used to call SNPs and genotypes for each of the 44 individuals based on BAM files, separately for each DPI. For each BAM file, reads with a mapping quality less than 15 were discarded using SAMtools^49^. Only bases called with a quality above 15 were used to calculate the coverage at each base position. Further, we followed strict criteria in VarScan to call heterozygous SNPs with high confidence (–min-reads2 10 –min-freq-for-hom 0.9 –min-var-freq 0.1). The resulting VCF files per DPI, along with the corresponding BAM files were run through the WASP’s^50^ mappability pipeline for correcting allelic mapping biases and performing unbiased removal of duplicate reads. The corrected BAM files were used to call SNPs (using the same criteria as before) and the resulting VCF file was annotated using SnpEff v4.3^51^ and filtered to include only exonic SNPs within the candidate genes. Next, we used Beagle v4.0^52^ to phase the exonic SNPs with the significant *cis*-eQTL SNPs identified for each candidate gene. Finally, the ASE analysis was performed separately for each gene region and DPI using the ASEperRegion module (https://github.com/molgenis/systemsgenetics/wiki/Basic-Usage), which is part of the Molgenis systems genetics pipeline^53^. Briefly, this procedure involved two steps. First, individual corrected BAM files were read along with the genotype information file (phased VCF per candidate gene per DPI) to determine how many reads mapped to either the reference or alternative allele of heterozygous exonic SNPs of the *cis*-gene that was in phase with the *cis*-eQTL SNP being tested. Next, the ASE test was performed using maximum likelihood estimation in combination with a beta-binomial likelihood ratio test (LRT). Over-dispersion of allele-specific reads in the individual samples was also determined and accounted for while estimating the beta binomial maximum likelihood.

## Additional Information

**Accession codes:** The RNA-Seq data from PHGC3 trial is available from the NCBI SRA repository under BioProject accession PRJNA311061and the data from PHGC5 trial is available from NCBI GEO repository under Series accession GSE78762.

**How to cite this article**: Kommadath, A. *et al*. Genetic architecture of gene expression underlying variation in host response to porcine reproductive and respiratory syndrome virus infection. *Sci. Rep.* **7**, 46203; doi: 10.1038/srep46203 (2017).

**Publisher's note:** Springer Nature remains neutral with regard to jurisdictional claims in published maps and institutional affiliations.

## Supplementary Material

Supplementary Information

## Figures and Tables

**Table 1:** Candidate *cis*-genes that overlap with GWAS regions for VL or WG.

Trait	Chr:Position (in Mb)	*cis*-genes (Nr. of *cis*-markers)
VL	4:99	*FCRL6* (1), *FCER1A* (1), *CD1D* (1)
	4:139	*GBP5* (8), *GBP6* (1)
WG	1:123	*ICE2* (2)
	3:138	*CMPK2* (1)
	4:139	*GBP5* (7)
	7:27	*CCHCR1* (1), *PPT2* (1)

Supplementary information,

Supplementary Information.pdf.
