# Supplementary material for: Genetic architecture of gene expression underlying variation in host response to porcine reproductive and respiratory syndrome virus infection
Source: Sci Rep. 2017 Apr 10;7:46203. doi: 10.1038/srep46203 (PMC5385538; doi:10.1038/srep46203)
Supplement: Supplementary Information [file srep46203-s1.pdf]

Genetic architecture of gene expression underlying variation in host response to porcine reproductive and respiratory syndrome virus infection

Arun Kommadath[1†], Hua Bao[1,7†], Igseo Choi[2], James M. Reecy[3], James E. Koltes[3,4], Elyn Fritz-Waters[3], Chris J. Eisley[3,5], Jason R. Grant[1], Robert R.R. Rowland[6], Christopher K. Tuggle[3], Jack C.M. Dekkers[3], Joan K. Lunney[2], Le Luo Guan[1], Paul Stothard[1*], Graham S. Plastow[1*]

[†] These authors contributed equally to this work

[*] Corresponding authors: Graham S. Plastow (plastow@ualberta.ca) and Paul Stothard (stothard@ualberta.ca)

Supplementary Figure S1. PCA plot of genotypes from 44 pigs

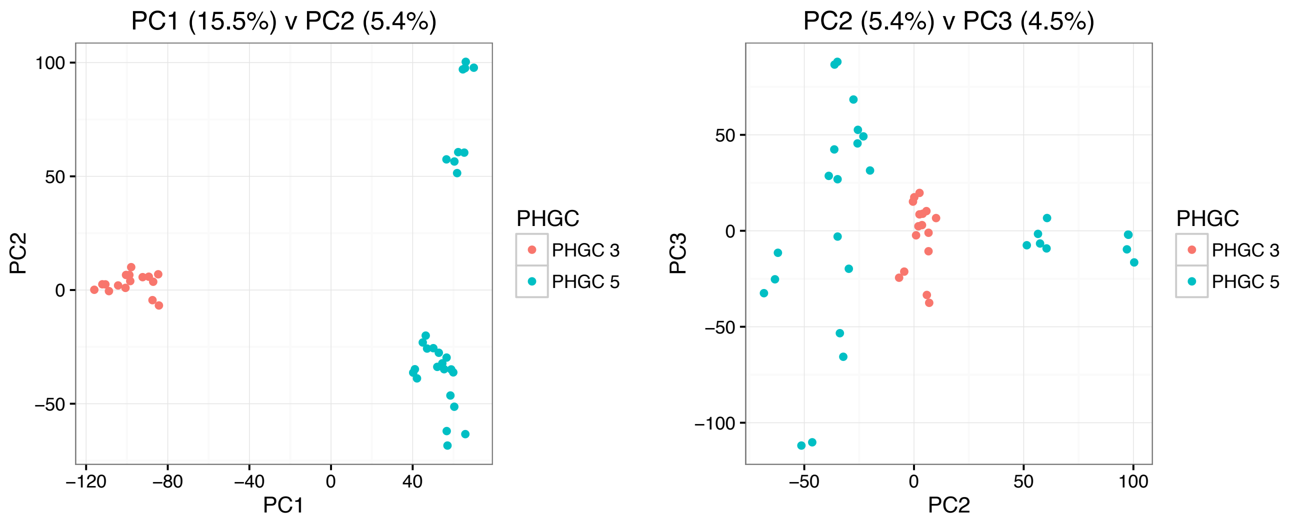

Supplementary Figure S2. PCA plot of expression levels from 190 samples (44 pigs)

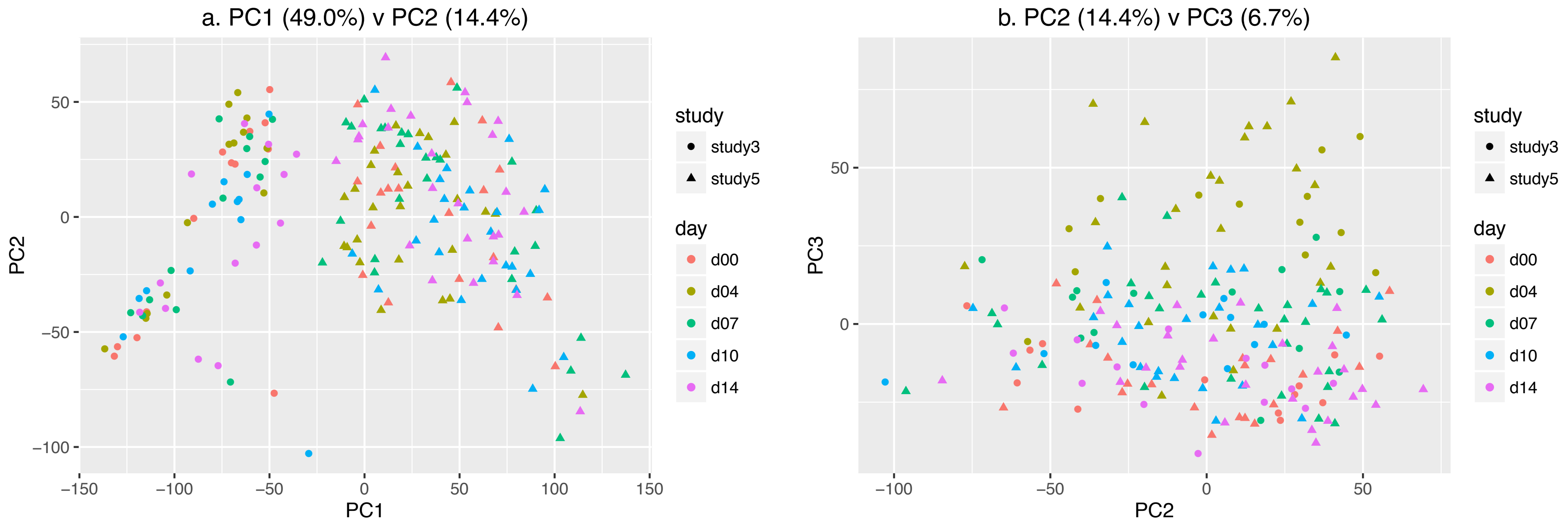

Supplementary Table S1A. Detailed information on the experimental pigs and their serum viral load

Animal_ID	Trial	Gender	Farrow Date	Viral load at different days post infection (DPI)								
				0	4	7	11	14	21	28	35	42
3031	PHGC3	M	09-03-11	0	6.00407	6.33162	5.57903	4.53626	2.87155	3.4474	1.55808	3.19469
3056	PHGC3	M	09-03-14	0	6.29044	6.57797	6.02605	5.82613	2.42912	1.93893	1.57961	1.47851
3068	PHGC3	M	09-03-11	0	6.45382	6.58947	6.22412	6.5322	0	1.10909	4.7758	0
3074	PHGC3	M	09-03-11	0	4.7327	5.73065	5.94774	6.91566	4.98191	2.84281	1.31752	0
3089	PHGC3	M	09-03-14	0	6.60157	7.03836	5.46721	4.99416	3.50383	2.30162	1.99503	0
3094	PHGC3	M	09-03-11	0	6.23359	6.2907	6.21218	5.8723	3.02399	4.52373	0.69375	0
3101	PHGC3	M	09-03-14	0	6.24281	6.60798	6.05093	5.996	2.59116	3.08262	1.23757	0.32368
3112	PHGC3	M	09-03-14	0	6.80333	7.05707	6.06637	6.16309	3.58656	2.42236	2.59828	1.07839
3113	PHGC3	M	09-03-14	0	5.98106	6.47378	5.46203	5.85883	2.77455	4.05888	0	2.00393
3122	PHGC3	M	09-03-16	0	6.3922	6.7958	6.42887	6.39704	5.20643	4.52133	1.87076	4.16829
3129	PHGC3	M	09-03-14	0	6.99792	6.86898	5.88658	4.95549	3.49115	4.13673	3.4638	1.92216
3148	PHGC3	M	09-03-16	0	5.47906	6.15202	6.49205	6.58403	5.21728	3.27299	3.03247	2.80753
3159	PHGC3	M	09-03-13	0	5.54521	6.39322	6.51299	6.28362	3.05286	4.36872	1.60992	2.16056
3170	PHGC3	M	09-03-13	0	5.55601	6.49127	5.49522	4.1934	2.34479	1.92338	2.98563	0.32068
3174	PHGC3	M	09-03-14	0	5.57233	6.51776	5.62506	5.11632	2.16499	3.10588	0	0.74597
3192	PHGC3	M	09-03-14	0	5.92967	6.39224	5.49398	4.89821	3.68993	4.46869	2.79281	0
5001	PHGC5	M	09-09-12	0	5.41807	6.41799	6.31812	5.82586	4.87458	4.10318	2.87721	0.49304
5002	PHGC5	M	09-09-11	0	4.83013	6.12183	6.17545	5.98294	4.54531	5.56332	3.94998	0.32883
5004	PHGC5	F	09-09-12	0	5.78283	6.24735	5.82956	5.00062	3.76461	5.60609	0.85084	1.27598
5005	PHGC5	M	09-09-11	0	5.63389	6.02592	6.0228	5.63553	4.43391	3.13415	0.69989	0
5019	PHGC5	F	09-09-11	0	4.952	6.03973	6.1825	5.32764	5.06375	2.16077	NA	0.7839
5027	PHGC5	M	09-09-12	0	5.93233	5.99566	5.02244	4.90666	2.73239	1.87911	4.06783	0
5028	PHGC5	F	09-09-11	0	4.63501	6.22889	5.91172	5.81216	3.67178	3.18324	1.80648	0
5033	PHGC5	M	09-09-12	0	5.69658	5.99376	5.33363	4.75611	4.73481	3.23958	0	0
5041	PHGC5	F	09-09-11	0	5.22194	6.2556	6.12751	5.57606	4.13808	3.35694	2.18822	0.49091
5081	PHGC5	F	09-09-12	0	5.47387	6.32539	6.29314	5.8819	3.11054	2.9246	2.62554	0
5082	PHGC5	M	09-09-11	0	4.61805	5.92256	5.74466	5.679	3.51041	3.20008	1.97825	0.91374
5089	PHGC5	F	09-09-12	0	5.76971	5.72529	4.94575	4.33856	1.58715	1.26352	1.30684	2.01786
5090	PHGC5	F	09-09-12	0	5.29353	6.24049	6.01055	5.97379	4.01899	2.87679	2.79391	0
5119	PHGC5	F	09-09-12	0	5.96315	6.19244	4.79293	3.81659	1.37066	0.37597	0	0
5142	PHGC5	F	09-09-11	0	5.67504	5.71785	5.70831	4.77526	3.837	3.46475	0	0.21388

5155	PHGC5	F	09-09-12	0	6.09158	6.13678	5.42497	4.71671	3.18552	3.10595	1.07592	1.4923
5178	PHGC5	F	09-09-11	0	6.96072	6.10568	6.18302	5.77613	4.87701	4.08846	0.62806	0
5145	PHGC5	F	09-09-12	0	6.33265	6.26279	6.01051	5.73836	3.51606	2.2208	1.81042	1.00221
5159	PHGC5	F	09-09-12	0	6.44178	5.64635	5.6447	4.84992	2.10816	2.06553	3.19839	0.69382
5170	PHGC5	M	09-09-12	0	6.26143	6.04059	4.9114	4.22691	1.90442	4.5159	0.53368	NA
5186	PHGC5	F	09-09-11	0	5.8972	6.36077	5.92806	5.53839	3.39722	1.5521	1.08895	1.15388
5009	PHGC5	F	09-09-12	0	5.41418	5.9972	5.66796	5.78216	4.51092	4.06702	0	0
5026	PHGC5	F	09-09-12	0	5.28938	5.89214	5.79028	4.81209	3.03797	3.12579	3.68296	1.98643
5035	PHGC5	M	09-09-12	0	5.66219	6.41129	5.30741	4.30413	2.8828	1.61882	0	0
5036	PHGC5	M	09-09-11	0	4.48493	5.7076	6.12122	5.8595	4.64559	2.86403	0.74797	1.44476
5037	PHGC5	F	09-09-12	0	5.58597	5.79885	5.28997	4.30628	2.6165	1.87818	1.9434	0
5059	PHGC5	F	09-09-12	0	5.49502	5.97111	6.20331	5.64294	4.91866	1.07433	3.72363	0
5072	PHGC5	M	09-09-12	0	5.92377	5.98129	5.83444	4.88201	4.76495	3.62338	3.15448	0.82843

Supplementary Table S1B. Detailed information on the experimental pigs with body weights

Animal_ID	Trial	Gender	Farrow Date	Body weight at different days post infection (DPI)						
				0	7	14	21	28	35	40
3031	PHGC3	M	09-03-11	6.98532	8.70897	10.97694	14.60567	18.14369	20.41166	24.0404
3056	PHGC3	M	09-03-14	7.16676	8.61826	12.51915	15.05927	16.32933	22.67962	25.85477
3068	PHGC3	M	09-03-11	7.89251	9.7976	11.15837	13.78921	18.59729	21.31884	24.94758
3074	PHGC3	M	09-03-11	5.8967	8.52754	10.16047	11.61196	11.7934	16.78292	19.05088
3089	PHGC3	M	09-03-14	7.3482	7.71107	8.25538	10.25119	11.7934	15.42214	15.42214
3094	PHGC3	M	09-03-11	8.25538	9.61616	10.52334	14.33352	15.42214	20.86525	24.0404
3101	PHGC3	M	09-03-14	6.44101	7.62035	9.25328	11.43053	13.15418	19.50447	18.14369
3112	PHGC3	M	09-03-14	6.98532	7.3482	9.07185	12.51915	17.23651	19.95806	24.94758
3113	PHGC3	M	09-03-14	6.8946	8.25538	8.98113	10.16047	13.15418	14.96855	24.0404
3122	PHGC3	M	09-03-16	4.71736	5.71526	6.16886	5.44311	5.8967	7.71107	9.07185
3129	PHGC3	M	09-03-14	6.44101	7.80179	8.07394	9.61616	12.24699	14.96855	18.59729
3148	PHGC3	M	09-03-16	2.17724	3.81018	5.08023	6.35029	7.71107	9.52544	11.7934
3159	PHGC3	M	09-03-13	6.71317	9.344	11.52125	11.70268	14.96855	21.31884	24.49399
3170	PHGC3	M	09-03-13	7.25748	9.43472	11.7934	15.78501	18.59729	23.5868	28.57632
3174	PHGC3	M	09-03-14	7.62035	9.52544	11.52125	14.06136	19.50447	22.67962	26.30836
3192	PHGC3	M	09-03-14	6.71317	8.52754	9.344	6.35029	15.42214	17.23651	19.95806
5001	PHGC5	M	09-09-12	6.80389	9.344	11.7934	13.60777	18.14369	21.77243	24.49399
5002	PHGC5	M	09-09-11	5.08023	6.75853	8.89041	10.7955	13.60777	16.32933	19.50447
5004	PHGC5	F	09-09-12	7.16676	9.7976	13.06346	16.05717	20.41166	23.5868	25.85477
5005	PHGC5	M	09-09-11	6.35029	8.25538	10.52334	13.2449	16.78292	21.31884	23.5868
5019	PHGC5	F	09-09-11	7.3482	9.25328	13.42633	16.23861	21.31884	26.30836	27.66913
5027	PHGC5	M	09-09-12	9.16257	9.88831	12.88202	15.6943	19.95806	22.67962	25.40117
5028	PHGC5	F	09-09-11	6.53173	7.80179	11.33981	14.42424	18.14369	20.41166	24.94758
5033	PHGC5	M	09-09-12	7.89251	8.89041	9.88831	12.7913	16.78292	19.95806	23.13321
5041	PHGC5	F	09-09-11	6.21422	9.07185	12.97274	14.69639	18.59729	22.22603	26.76195
5081	PHGC5	F	09-09-12	8.07394	10.25119	13.33562	15.2407	18.14369	20.41166	24.94758
5082	PHGC5	M	09-09-11	6.62245	8.98113	10.16047	13.60777	17.6901	21.31884	27.21554
5089	PHGC5	F	09-09-12	6.71317	8.79969	11.15837	14.96855	19.95806	23.13321	28.57632
5090	PHGC5	F	09-09-12	6.71317	8.98113	10.52334	12.60987	16.78292	20.41166	23.5868
5119	PHGC5	F	09-09-12	8.70897	9.7976	12.70059	16.78292	19.50447	24.94758	22.67962

5142	PHGC5	F	09-09-11	7.16676	9.70688	12.7913	16.23861	21.31884	24.49399	28.57632
5155	PHGC5	F	09-09-12	7.43891	9.52544	13.42633	17.87154	21.31884	25.40117	29.9371
5178	PHGC5	F	09-09-11	7.80179	9.25328	12.24699	14.69639	19.50447	21.77243	26.30836
5145	PHGC5	F	09-09-12	6.44101	8.61826	10.7955	14.33352	19.05088	24.0404	28.12273
5159	PHGC5	F	09-09-12	7.07604	10.06975	13.2449	17.41795	21.77243	27.21554	30.39069
5170	PHGC5	M	09-09-12	7.52963	8.79969	12.7913	16.60148	20.41166	25.40117	28.12273
5186	PHGC5	F	09-09-11	7.16676	9.61616	12.97274	16.96435	20.86525	25.40117	26.30836
5009	PHGC5	F	09-09-12	6.80389	7.71107	9.88831	12.42843	16.78292	20.86525	23.13321
5026	PHGC5	F	09-09-12	6.44101	8.16466	10.88622	14.96855	18.14369	19.95806	26.30836
5035	PHGC5	M	09-09-12	8.21002	11.61196	15.78501	19.77663	21.31884	26.76195	32.65865
5036	PHGC5	M	09-09-11	6.1235	8.52754	11.83876	13.97064	21.31884	19.05088	24.94758
5037	PHGC5	F	09-09-12	6.03278	8.52754	11.52125	14.42424	17.23651	20.86525	26.76195
5059	PHGC5	F	09-09-12	7.25748	10.43262	12.97274	15.78501	19.50447	23.13321	27.21554
5072	PHGC5	M	09-09-12	6.71317	7.80179	8.25538	7.1214	7.71107	9.97903	11.7934

Supplementary Table S2. Complete list of enriched GO terms per cluster

Cluster	ID	Description	GeneRatio (hits in gene list)	BackgroundRatio (hits in population)	pvalue	Benjamini-Hochberg adjusted pvalue	qvalue (False discovery rate)
C1	GO:0006952	defense response	34/259	265/5979	2.31E-08	4.53E-05	3.97E-05
C1	GO:0006955	immune response	38/259	332/5979	6.12E-08	5.98E-05	1.05E-04
C1	GO:0006954	inflammatory response	19/259	161/5979	1.76E-04	1.09E-01	3.02E-01
C1	GO:0006691	leukotriene metabolic process	5/259	10/5979	5.79E-04	2.47E-01	9.88E-01
C1	GO:0043449	cellular alkene metabolic process	5/259	10/5979	5.79E-04	2.47E-01	9.88E-01
C1	GO:0046365	monosaccharide catabolic process	8/259	36/5979	7.31E-04	2.49E-01	1.25E+00
C1	GO:0051050	positive regulation of transport	13/259	97/5979	8.71E-04	2.47E-01	1.48E+00
C1	GO:0009611	response to wounding	22/259	236/5979	1.19E-03	2.84E-01	2.03E+00
C1	GO:0002703	regulation of leukocyte mediated immunity	8/259	40/5979	1.41E-03	2.92E-01	2.39E+00
C1	GO:0002697	regulation of immune effector process	10/259	65/5979	1.73E-03	3.14E-01	2.93E+00
C1	GO:0046164	alcohol catabolic process	8/259	42/5979	1.89E-03	3.10E-01	3.20E+00
C1	GO:0006690	icosanoid metabolic process	6/259	22/5979	2.07E-03	3.08E-01	3.49E+00
C1	GO:0042742	defense response to bacterium	7/259	32/5979	2.14E-03	2.95E-01	3.61E+00
C1	GO:0051047	positive regulation of secretion	8/259	43/5979	2.18E-03	2.80E-01	3.67E+00
C1	GO:0016052	carbohydrate catabolic process	9/259	55/5979	2.25E-03	2.70E-01	3.79E+00
C1	GO:0044275	cellular carbohydrate catabolic process	8/259	44/5979	2.50E-03	2.78E-01	4.20E+00
C1	GO:0001817	regulation of cytokine production	13/259	111/5979	2.82E-03	2.92E-01	4.73E+00
C1	GO:0019320	hexose catabolic process	7/259	34/5979	2.96E-03	2.89E-01	4.95E+00
C1	GO:0006631	fatty acid metabolic process	12/259	99/5979	3.38E-03	3.08E-01	5.64E+00
C1	GO:0019318	hexose metabolic process	13/259	114/5979	3.52E-03	3.05E-01	5.87E+00
C1	GO:0033559	unsaturated fatty acid metabolic process	6/259	25/5979	3.76E-03	3.08E-01	6.25E+00
C1	GO:0019370	leukotriene biosynthetic process	4/259	8/5979	3.78E-03	2.98E-01	6.29E+00
C1	GO:0050766	positive regulation of phagocytosis	4/259	8/5979	3.78E-03	2.98E-01	6.29E+00
C1	GO:0043450	alkene biosynthetic process	4/259	8/5979	3.78E-03	2.98E-01	6.29E+00
C1	GO:0005996	monosaccharide metabolic process	14/259	132/5979	4.34E-03	3.21E-01	7.19E+00
C1	GO:0042110	T cell activation	11/259	89/5979	4.72E-03	3.31E-01	7.79E+00
C1	GO:0015849	organic acid transport	8/259	50/5979	5.23E-03	3.48E-01	8.60E+00
C1	GO:0046942	carboxylic acid transport	8/259	50/5979	5.23E-03	3.48E-01	8.60E+00
C1	GO:0002699	positive regulation of immune effector process	6/259	28/5979	6.25E-03	3.88E-01	1.02E+01
C1	GO:0002263	cell activation during immune response	6/259	29/5979	7.30E-03	4.24E-01	1.18E+01

C1	GO:0002366	leukocyte activation during immune response	6/259	29/5979	7.30E-03	4.24E-01	1.18E+01
C1	GO:0050764	regulation of phagocytosis	4/259	10/5979	7.60E-03	4.25E-01	1.23E+01
C1	GO:0002886	regulation of myeloid leukocyte mediated immunity	4/259	10/5979	7.60E-03	4.25E-01	1.23E+01
C1	GO:0048584	positive regulation of response to stimulus	13/259	126/5979	7.87E-03	4.24E-01	1.27E+01
C1	GO:0045321	leukocyte activation	15/259	158/5979	7.99E-03	4.18E-01	1.29E+01
C1	GO:0002705	positive regulation of leukocyte mediated immunity	5/259	20/5979	9.50E-03	4.63E-01	1.51E+01
C1	GO:0002708	positive regulation of lymphocyte mediated immunity	5/259	20/5979	9.50E-03	4.63E-01	1.51E+01
C1	GO:0001775	cell activation	16/259	178/5979	9.62E-03	4.57E-01	1.53E+01
C1	GO:0006007	glucose catabolic process	6/259	31/5979	9.73E-03	4.50E-01	1.54E+01
C1	GO:0032103	positive regulation of response to external stimulus	6/259	31/5979	9.73E-03	4.50E-01	1.54E+01
C2	GO:0006259	DNA metabolic process	165/1864	340/5979	9.44E-12	3.66E-08	1.75E-08
C2	GO:0006260	DNA replication	73/1864	133/5979	2.33E-08	4.51E-05	4.31E-05
C2	GO:0006281	DNA repair	101/1864	206/5979	8.63E-08	1.12E-04	1.60E-04
C2	GO:0000279	M phase	109/1864	233/5979	5.18E-07	5.02E-04	9.59E-04
C2	GO:0022403	cell cycle phase	131/1864	293/5979	7.56E-07	5.86E-04	1.40E-03
C2	GO:0006974	response to DNA damage stimulus	119/1864	265/5979	2.00E-06	1.29E-03	3.71E-03
C2	GO:0007067	mitosis	85/1864	176/5979	2.29E-06	1.26E-03	4.23E-03
C2	GO:0000280	nuclear division	85/1864	176/5979	2.29E-06	1.26E-03	4.23E-03
C2	GO:0006261	DNA-dependent DNA replication	28/1864	41/5979	4.77E-06	2.31E-03	8.83E-03
C2	GO:0051301	cell division	100/1864	218/5979	4.81E-06	2.07E-03	8.91E-03
C2	GO:0000087	M phase of mitotic cell cycle	85/1864	179/5979	5.33E-06	2.06E-03	9.86E-03
C2	GO:0048285	organelle fission	86/1864	183/5979	7.76E-06	2.73E-03	1.44E-02
C2	GO:0007049	cell cycle	205/1864	522/5979	3.53E-05	1.14E-02	6.54E-02
C2	GO:0006289	nucleotide-excision repair	30/1864	49/5979	4.38E-05	1.30E-02	8.10E-02
C2	GO:0006695	cholesterol biosynthetic process	14/1864	17/5979	1.56E-04	4.23E-02	2.88E-01
C2	GO:0055114	oxidation reduction	141/1864	353/5979	2.98E-04	7.40E-02	5.50E-01
C2	GO:0022402	cell cycle process	154/1864	391/5979	3.29E-04	7.67E-02	6.08E-01
C2	GO:0007051	spindle organization	24/1864	40/5979	4.85E-04	1.05E-01	8.94E-01
C2	GO:0006310	DNA recombination	36/1864	69/5979	5.14E-04	1.05E-01	9.47E-01
C2	GO:0006412	translation	97/1864	233/5979	6.05E-04	1.16E-01	1.12E+00
C2	GO:0008203	cholesterol metabolic process	28/1864	50/5979	6.32E-04	1.15E-01	1.16E+00
C2	GO:0016126	sterol biosynthetic process	16/1864	23/5979	7.81E-04	1.34E-01	1.44E+00
C2	GO:0000070	mitotic sister chromatid segregation	19/1864	30/5979	1.00E-03	1.62E-01	1.84E+00
C2	GO:0000226	microtubule cytoskeleton organization	41/1864	84/5979	1.08E-03	1.67E-01	1.98E+00
C2	GO:0033554	cellular response to stress	144/1864	371/5979	1.09E-03	1.61E-01	1.99E+00
C2	GO:0000278	mitotic cell cycle	113/1864	283/5979	1.29E-03	1.82E-01	2.36E+00

C2	GO:0000819	sister chromatid segregation	19/1864	31/5979	1.70E-03	2.25E-01	3.11E+00
C2	GO:0006284	base-excision repair	14/1864	20/5979	1.86E-03	2.34E-01	3.38E+00
C2	GO:0051276	chromosome organization	123/1864	315/5979	2.03E-03	2.45E-01	3.70E+00
C2	GO:0016125	sterol metabolic process	29/1864	56/5979	2.40E-03	2.74E-01	4.35E+00
C2	GO:0007017	microtubule-based process	57/1864	130/5979	2.52E-03	2.78E-01	4.56E+00
C2	GO:0006694	steroid biosynthetic process	21/1864	38/5979	4.65E-03	4.42E-01	8.27E+00
C2	GO:0022613	ribonucleoprotein complex biogenesis	61/1864	145/5979	5.41E-03	4.82E-01	9.56E+00
C2	GO:0006414	translational elongation	32/1864	67/5979	6.46E-03	5.33E-01	1.13E+01
C2	GO:0032200	telomere organization	15/1864	25/5979	8.55E-03	6.24E-01	1.47E+01
C2	GO:0008202	steroid metabolic process	39/1864	87/5979	8.92E-03	6.29E-01	1.53E+01
C3	GO:0009611	response to wounding	116/1665	236/5979	2.93E-12	1.24E-08	5.48E-09
C3	GO:0043067	regulation of programmed cell death	181/1665	454/5979	9.93E-09	2.11E-05	1.86E-05
C3	GO:0042981	regulation of apoptosis	180/1665	451/5979	9.99E-09	1.41E-05	1.87E-05
C3	GO:0010941	regulation of cell death	181/1665	456/5979	1.47E-08	1.56E-05	2.74E-05
C3	GO:0010033	response to organic substance	144/1665	347/5979	2.20E-08	1.87E-05	4.12E-05
C3	GO:0016192	vesicle-mediated transport	147/1665	356/5979	2.26E-08	1.60E-05	4.22E-05
C3	GO:0016044	membrane organization	102/1665	231/5979	9.61E-08	5.83E-05	1.80E-04
C3	GO:0010627	regulation of protein kinase cascade	70/1665	143/5979	1.22E-07	6.49E-05	2.29E-04
C3	GO:0006952	defense response	113/1665	265/5979	1.71E-07	8.07E-05	3.20E-04
C3	GO:0006955	immune response	135/1665	332/5979	2.71E-07	1.15E-04	5.06E-04
C3	GO:0007166	cell surface receptor linked signal transduction	176/1665	459/5979	4.09E-07	1.58E-04	7.64E-04
C3	GO:0050878	regulation of body fluid levels	34/1665	55/5979	5.41E-07	1.91E-04	1.01E-03
C3	GO:0007243	protein kinase cascade	94/1665	215/5979	5.68E-07	1.85E-04	1.06E-03
C3	GO:0043066	negative regulation of apoptosis	86/1665	193/5979	7.02E-07	2.13E-04	1.31E-03
C3	GO:0002237	response to molecule of bacterial origin	31/1665	49/5979	9.69E-07	2.74E-04	1.81E-03
C3	GO:0006954	inflammatory response	74/1665	161/5979	1.14E-06	3.03E-04	2.14E-03
C3	GO:0043069	negative regulation of programmed cell death	86/1665	195/5979	1.19E-06	2.97E-04	2.23E-03
C3	GO:0009615	response to virus	32/1665	52/5979	1.46E-06	3.44E-04	2.73E-03
C3	GO:0060548	negative regulation of cell death	86/1665	196/5979	1.54E-06	3.44E-04	2.88E-03
C3	GO:0042060	wound healing	41/1665	74/5979	1.58E-06	3.34E-04	2.95E-03
C3	GO:0007242	intracellular signaling cascade	234/1665	654/5979	2.39E-06	4.84E-04	4.48E-03
C3	GO:0010647	positive regulation of cell communication	71/1665	156/5979	3.02E-06	5.82E-04	5.64E-03
C3	GO:0012501	programmed cell death	143/1665	369/5979	3.05E-06	5.63E-04	5.71E-03
C3	GO:0050817	coagulation	28/1665	44/5979	3.20E-06	5.65E-04	5.98E-03
C3	GO:0007596	blood coagulation	28/1665	44/5979	3.20E-06	5.65E-04	5.98E-03
C3	GO:0043122	regulation of I-kappaB kinase/NF-kappaB cascade	41/1665	76/5979	3.85E-06	6.53E-04	7.19E-03

C3	GO:0007599	hemostasis	30/1665	49/5979	3.92E-06	6.40E-04	7.33E-03
C3	GO:0016265	death	162/1665	432/5979	6.04E-06	9.49E-04	1.13E-02
C3	GO:0032496	response to lipopolysaccharide	27/1665	43/5979	7.18E-06	1.09E-03	1.34E-02
C3	GO:0008219	cell death	161/1665	430/5979	7.19E-06	1.05E-03	1.34E-02
C3	GO:0006915	apoptosis	139/1665	362/5979	7.59E-06	1.07E-03	1.42E-02
C3	GO:0010740	positive regulation of protein kinase cascade	47/1665	95/5979	1.39E-05	1.90E-03	2.60E-02
C3	GO:0009967	positive regulation of signal transduction	66/1665	148/5979	1.61E-05	2.13E-03	3.00E-02
C3	GO:0010324	membrane invagination	59/1665	129/5979	1.96E-05	2.52E-03	3.67E-02
C3	GO:0006897	endocytosis	59/1665	129/5979	1.96E-05	2.52E-03	3.67E-02
C3	GO:0015918	sterol transport	11/1665	11/5979	2.25E-05	2.81E-03	4.21E-02
C3	GO:0030301	cholesterol transport	11/1665	11/5979	2.25E-05	2.81E-03	4.21E-02
C3	GO:0043068	positive regulation of programmed cell death	105/1665	265/5979	2.60E-05	3.15E-03	4.86E-02
C3	GO:0009617	response to bacterium	39/1665	76/5979	3.12E-05	3.67E-03	5.83E-02
C3	GO:0010942	positive regulation of cell death	105/1665	266/5979	3.13E-05	3.58E-03	5.84E-02
C3	GO:0043065	positive regulation of apoptosis	104/1665	264/5979	3.78E-05	4.21E-03	7.07E-02
C3	GO:0006928	cell motion	76/1665	181/5979	4.37E-05	4.75E-03	8.17E-02
C3	GO:0051051	negative regulation of transport	34/1665	64/5979	4.65E-05	4.93E-03	8.70E-02
C3	GO:0032101	regulation of response to external stimulus	35/1665	67/5979	5.48E-05	5.66E-03	1.03E-01
C3	GO:0006916	anti-apoptosis	55/1665	122/5979	6.39E-05	6.44E-03	1.19E-01
C3	GO:0060341	regulation of cellular localization	48/1665	104/5979	1.02E-04	9.98E-03	1.90E-01
C3	GO:0051046	regulation of secretion	37/1665	74/5979	1.04E-04	1.00E-02	1.95E-01
C3	GO:0044093	positive regulation of molecular function	113/1665	298/5979	1.12E-04	1.05E-02	2.09E-01
C3	GO:0030168	platelet activation	13/1665	16/5979	1.18E-04	1.08E-02	2.20E-01
C3	GO:0051603	proteolysis involved in cellular protein catabolic process	144/1665	397/5979	1.42E-04	1.27E-02	2.65E-01
C3	GO:0010876	lipid localization	34/1665	67/5979	1.49E-04	1.31E-02	2.78E-01
C3	GO:0044257	cellular protein catabolic process	144/1665	398/5979	1.62E-04	1.40E-02	3.03E-01
C3	GO:0032880	regulation of protein localization	35/1665	70/5979	1.68E-04	1.41E-02	3.13E-01
C3	GO:0016477	cell migration	48/1665	106/5979	1.78E-04	1.47E-02	3.33E-01
C3	GO:0048584	positive regulation of response to stimulus	55/1665	126/5979	1.81E-04	1.46E-02	3.37E-01
C3	GO:0034097	response to cytokine stimulus	26/1665	47/5979	2.03E-04	1.61E-02	3.79E-01
C3	GO:0043123	positive regulation of I-kappaB kinase/NF-kappaB cascade	34/1665	68/5979	2.13E-04	1.66E-02	3.97E-01
C3	GO:0007264	small GTPase mediated signal transduction	71/1665	174/5979	2.29E-04	1.75E-02	4.28E-01
C3	GO:0070201	regulation of establishment of protein localization	32/1665	63/5979	2.40E-04	1.80E-02	4.47E-01
C3	GO:0006508	proteolysis	187/1665	542/5979	3.09E-04	2.27E-02	5.76E-01
C3	GO:0051674	localization of cell	49/1665	111/5979	3.15E-04	2.28E-02	5.88E-01
C3	GO:0048870	cell motility	49/1665	111/5979	3.15E-04	2.28E-02	5.88E-01

C3	GO:0009725	response to hormone stimulus	63/1665	152/5979	3.31E-04	2.35E-02	6.17E-01
C3	GO:0051050	positive regulation of transport	44/1665	97/5979	3.34E-04	2.33E-02	6.22E-01
C3	GO:0007169	transmembrane receptor protein tyrosine kinase signaling pathway	40/1665	86/5979	3.55E-04	2.44E-02	6.62E-01
C3	GO:0019941	modification-dependent protein catabolic process	136/1665	379/5979	3.81E-04	2.58E-02	7.10E-01
C3	GO:0043632	modification-dependent macromolecule catabolic process	136/1665	379/5979	3.81E-04	2.58E-02	7.10E-01
C3	GO:0030163	protein catabolic process	145/1665	408/5979	3.90E-04	2.59E-02	7.27E-01
C3	GO:0051091	positive regulation of transcription factor activity	21/1665	36/5979	4.12E-04	2.70E-02	7.67E-01
C3	GO:0009719	response to endogenous stimulus	69/1665	171/5979	4.15E-04	2.68E-02	7.74E-01
C3	GO:0051223	regulation of protein transport	31/1665	62/5979	4.35E-04	2.76E-02	8.10E-01
C3	GO:0002274	myeloid leukocyte activation	18/1665	29/5979	4.80E-04	3.00E-02	8.95E-01
C3	GO:0007155	cell adhesion	76/1665	193/5979	4.87E-04	3.00E-02	9.07E-01
C3	GO:0022610	biological adhesion	76/1665	193/5979	4.87E-04	3.00E-02	9.07E-01
C3	GO:0008284	positive regulation of cell proliferation	74/1665	187/5979	4.94E-04	2.99E-02	9.19E-01
C3	GO:0006869	lipid transport	30/1665	60/5979	5.52E-04	3.29E-02	1.03E+00
C3	GO:0016337	cell-cell adhesion	30/1665	60/5979	5.52E-04	3.29E-02	1.03E+00
C3	GO:0007186	G-protein coupled receptor protein signaling pathway	59/1665	143/5979	6.04E-04	3.55E-02	1.12E+00
C3	GO:0001501	skeletal system development	36/1665	77/5979	6.65E-04	3.85E-02	1.24E+00
C3	GO:0048545	response to steroid hormone stimulus	32/1665	66/5979	6.71E-04	3.83E-02	1.25E+00
C3	GO:0043085	positive regulation of catalytic activity	96/1665	257/5979	7.09E-04	3.99E-02	1.32E+00
C3	GO:0048878	chemical homeostasis	72/1665	183/5979	7.19E-04	3.99E-02	1.34E+00
C3	GO:0002221	pattern recognition receptor signaling pathway	11/1665	14/5979	8.62E-04	4.70E-02	1.60E+00
C3	GO:0042592	homeostatic process	116/1665	322/5979	9.18E-04	4.94E-02	1.70E+00
C3	GO:0010927	cellular component assembly involved in morphogenesis	10/1665	12/5979	9.20E-04	4.89E-02	1.71E+00
C3	GO:0019221	cytokine-mediated signaling pathway	23/1665	43/5979	9.73E-04	5.10E-02	1.80E+00
C3	GO:0031349	positive regulation of defense response	23/1665	43/5979	9.73E-04	5.10E-02	1.80E+00
C3	GO:0051047	positive regulation of secretion	23/1665	43/5979	9.73E-04	5.10E-02	1.80E+00
C3	GO:0051094	positive regulation of developmental process	51/1665	122/5979	1.07E-03	5.51E-02	1.98E+00
C3	GO:0006909	phagocytosis	19/1665	33/5979	1.07E-03	5.45E-02	1.98E+00
C3	GO:0007249	I-kappaB kinase/NF-kappaB cascade	24/1665	46/5979	1.13E-03	5.69E-02	2.09E+00
C3	GO:0051240	positive regulation of multicellular organismal process	45/1665	105/5979	1.22E-03	6.06E-02	2.26E+00
C3	GO:0051241	negative regulation of multicellular organismal process	28/1665	57/5979	1.25E-03	6.13E-02	2.31E+00
C3	GO:0032103	positive regulation of response to external stimulus	18/1665	31/5979	1.35E-03	6.55E-02	2.50E+00
C3	GO:0051092	positive regulation of NF-kappaB transcription factor activity	15/1665	24/5979	1.63E-03	7.74E-02	3.01E+00
C3	GO:0045428	regulation of nitric oxide biosynthetic process	12/1665	17/5979	1.64E-03	7.68E-02	3.02E+00
C3	GO:0001944	vasculature development	43/1665	101/5979	1.87E-03	8.62E-02	3.43E+00
C3	GO:0002758	innate immune response-activating signal transduction	11/1665	15/5979	1.94E-03	8.86E-02	3.57E+00

C3	GO:0002218	activation of innate immune response	11/1665	15/5979	1.94E-03	8.86E-02	3.57E+00
C3	GO:0006576	biogenic amine metabolic process	20/1665	37/5979	2.02E-03	9.09E-02	3.71E+00
C3	GO:0044265	cellular macromolecule catabolic process	164/1665	485/5979	2.08E-03	9.27E-02	3.82E+00
C3	GO:0007259	JAK-STAT cascade	16/1665	27/5979	2.18E-03	9.58E-02	4.00E+00
C3	GO:0042127	regulation of cell proliferation	123/1665	352/5979	2.30E-03	9.97E-02	4.21E+00
C3	GO:0051090	regulation of transcription factor activity	28/1665	59/5979	2.36E-03	1.01E-01	4.31E+00
C3	GO:0007167	enzyme linked receptor protein signaling pathway	52/1665	129/5979	2.43E-03	1.03E-01	4.45E+00
C3	GO:0033344	cholesterol efflux	7/1665	7/5979	2.46E-03	1.03E-01	4.51E+00
C3	GO:0032760	positive regulation of tumor necrosis factor production	9/1665	11/5979	2.52E-03	1.05E-01	4.61E+00
C3	GO:0032570	response to progesterone stimulus	9/1665	11/5979	2.52E-03	1.05E-01	4.61E+00
C3	GO:0051607	defense response to virus	9/1665	11/5979	2.52E-03	1.05E-01	4.61E+00
C3	GO:0051048	negative regulation of secretion	13/1665	20/5979	2.54E-03	1.04E-01	4.65E+00
C3	GO:0050727	regulation of inflammatory response	19/1665	35/5979	2.58E-03	1.05E-01	4.72E+00
C3	GO:0010743	regulation of foam cell differentiation	8/1665	9/5979	2.65E-03	1.07E-01	4.84E+00
C3	GO:0043388	positive regulation of DNA binding	22/1665	43/5979	2.66E-03	1.06E-01	4.87E+00
C3	GO:0051224	negative regulation of protein transport	15/1665	25/5979	2.77E-03	1.09E-01	5.05E+00
C3	GO:0001568	blood vessel development	42/1665	100/5979	2.88E-03	1.12E-01	5.24E+00
C3	GO:0007267	cell-cell signaling	51/1665	127/5979	2.95E-03	1.14E-01	5.38E+00
C3	GO:0006917	induction of apoptosis	75/1665	201/5979	3.00E-03	1.15E-01	5.47E+00
C3	GO:0012502	induction of programmed cell death	75/1665	201/5979	3.00E-03	1.15E-01	5.47E+00
C3	GO:0006575	cellular amino acid derivative metabolic process	34/1665	77/5979	3.11E-03	1.17E-01	5.67E+00
C3	GO:0001819	positive regulation of cytokine production	28/1665	60/5979	3.17E-03	1.18E-01	5.76E+00
C3	GO:0010648	negative regulation of cell communication	43/1665	104/5979	3.59E-03	1.32E-01	6.50E+00
C3	GO:0051101	regulation of DNA binding	31/1665	69/5979	3.64E-03	1.32E-01	6.59E+00
C3	GO:0043406	positive regulation of MAP kinase activity	22/1665	44/5979	3.78E-03	1.36E-01	6.84E+00
C3	GO:0051345	positive regulation of hydrolase activity	35/1665	81/5979	4.07E-03	1.45E-01	7.35E+00
C3	GO:0043523	regulation of neuron apoptosis	23/1665	47/5979	4.14E-03	1.45E-01	7.46E+00
C3	GO:0006643	membrane lipid metabolic process	23/1665	47/5979	4.14E-03	1.45E-01	7.46E+00
C3	GO:0051789	response to protein stimulus	28/1665	61/5979	4.20E-03	1.46E-01	7.57E+00
C3	GO:0006875	cellular metal ion homeostasis	28/1665	61/5979	4.20E-03	1.46E-01	7.57E+00
C3	GO:0055065	metal ion homeostasis	29/1665	64/5979	4.39E-03	1.51E-01	7.90E+00
C3	GO:0001817	regulation of cytokine production	45/1665	111/5979	4.41E-03	1.50E-01	7.93E+00
C3	GO:0030036	actin cytoskeleton organization	44/1665	108/5979	4.41E-03	1.49E-01	7.93E+00
C3	GO:0051099	positive regulation of binding	24/1665	50/5979	4.47E-03	1.50E-01	8.03E+00
C3	GO:0009057	macromolecule catabolic process	170/1665	514/5979	4.85E-03	1.60E-01	8.69E+00

C3	GO:0006665	sphingolipid metabolic process	22/1665	45/5979	5.26E-03	1.72E-01	9.40E+00
C3	GO:0030029	actin filament-based process	45/1665	112/5979	5.34E-03	1.73E-01	9.54E+00
C3	GO:0032870	cellular response to hormone stimulus	28/1665	62/5979	5.51E-03	1.76E-01	9.81E+00
C3	GO:0050818	regulation of coagulation	12/1665	19/5979	5.63E-03	1.78E-01	1.00E+01
C3	GO:0002224	toll-like receptor signaling pathway	9/1665	12/5979	5.72E-03	1.80E-01	1.02E+01
C3	GO:0030808	regulation of nucleotide biosynthetic process	14/1665	24/5979	5.72E-03	1.78E-01	1.02E+01
C3	GO:0030802	regulation of cyclic nucleotide biosynthetic process	14/1665	24/5979	5.72E-03	1.78E-01	1.02E+01
C3	GO:0050708	regulation of protein secretion	14/1665	24/5979	5.72E-03	1.78E-01	1.02E+01
C3	GO:0060191	regulation of lipase activity	17/1665	32/5979	6.29E-03	1.93E-01	1.11E+01
C3	GO:0050778	positive regulation of immune response	35/1665	83/5979	6.42E-03	1.95E-01	1.14E+01
C3	GO:0001775	cell activation	66/1665	178/5979	6.46E-03	1.95E-01	1.14E+01
C3	GO:0006874	cellular calcium ion homeostasis	26/1665	57/5979	6.65E-03	1.99E-01	1.17E+01
C3	GO:0010883	regulation of lipid storage	8/1665	10/5979	6.71E-03	1.99E-01	1.18E+01
C3	GO:0055074	calcium ion homeostasis	27/1665	60/5979	6.91E-03	2.03E-01	1.22E+01
C3	GO:0030855	epithelial cell differentiation	15/1665	27/5979	6.95E-03	2.02E-01	1.22E+01
C3	GO:0050714	positive regulation of protein secretion	11/1665	17/5979	7.11E-03	2.05E-01	1.25E+01
C3	GO:0032675	regulation of interleukin-6 production	13/1665	22/5979	7.33E-03	2.09E-01	1.29E+01
C3	GO:0070555	response to interleukin-1	7/1665	8/5979	7.52E-03	2.13E-01	1.32E+01
C3	GO:0045087	innate immune response	31/1665	72/5979	7.64E-03	2.14E-01	1.34E+01
C3	GO:0002253	activation of immune response	24/1665	52/5979	8.02E-03	2.22E-01	1.40E+01
C3	GO:0030799	regulation of cyclic nucleotide metabolic process	14/1665	25/5979	8.93E-03	2.43E-01	1.54E+01
C3	GO:0009968	negative regulation of signal transduction	39/1665	97/5979	9.72E-03	2.60E-01	1.67E+01
C3	GO:0051098	regulation of binding	38/1665	94/5979	9.77E-03	2.59E-01	1.68E+01
C3	GO:0001932	regulation of protein amino acid phosphorylation	34/1665	82/5979	9.79E-03	2.58E-01	1.68E+01
C3	GO:0000165	MAPKKK cascade	37/1665	91/5979	9.80E-03	2.57E-01	1.68E+01
C4	GO:0045449	regulation of transcription	190/664	1146/5979	3.79E-10	9.14E-07	6.66E-07
C4	GO:0006350	transcription	160/664	952/5979	6.74E-09	8.12E-06	1.18E-05
C4	GO:0006355	regulation of transcription, DNA-dependent	114/664	691/5979	5.25E-06	4.20E-03	9.21E-03
C4	GO:0051252	regulation of RNA metabolic process	117/664	716/5979	6.14E-06	3.69E-03	1.08E-02
C4	GO:0006468	protein amino acid phosphorylation	62/664	360/5979	3.67E-04	1.62E-01	6.43E-01
C4	GO:0007389	pattern specification process	17/664	71/5979	4.03E-03	8.02E-01	6.84E+00
C4	GO:0010717	regulation of epithelial to mesenchymal transition	4/664	4/5979	4.98E-03	8.21E-01	8.40E+00
C4	GO:0009952	anterior/posterior pattern formation	11/664	37/5979	5.64E-03	8.18E-01	9.46E+00
C4	GO:0017015	regulation of transforming growth factor beta receptor signaling pathway	7/664	17/5979	7.68E-03	8.73E-01	1.27E+01
C4	GO:0003002	regionalization	13/664	51/5979	8.44E-03	8.70E-01	1.38E+01

C4	GO:0010629	negative regulation of gene expression	43/664	265/5979	9.96E-03	8.88E-01	1.61E+01
C4	GO:0043473	pigmentation	8/664	23/5979	9.97E-03	8.66E-01	1.61E+01
C4	GO:0048339	paraxial mesoderm development	4/664	5/5979	1.14E-02	8.81E-01	1.83E+01
C4	GO:0007183	SMAD protein complex assembly	4/664	5/5979	1.14E-02	8.81E-01	1.83E+01
C4	GO:0007029	endoplasmic reticulum organization	5/664	9/5979	1.20E-02	8.74E-01	1.90E+01
C4	GO:0045767	regulation of anti-apoptosis	8/664	24/5979	1.28E-02	8.73E-01	2.02E+01
C4	GO:0007179	transforming growth factor beta receptor signaling pathway	8/664	24/5979	1.28E-02	8.73E-01	2.02E+01

Supplementary Table S3. Information on the top 10 windows (1 Mb each) that explained the greatest percentage of genetic variance for VL and WG *

Rank	GWAS regions for VL		GWAS regions for WG	
	Chr:position (in MB)	Genetic variance (%)	Chr:position (in MB)	Genetic variance (%)
1	4:139	13.2	4:139	9.14
2	X:113	1.24	5:72	2.61
3	1:292	0.70	7:27	1.45
4	9:13	0.64	10:67	0.94
5	X:112	0.49	4:7	0.84
6	9:47	0.48	11:1	0.71
7	9:29	0.46	3:138	0.56
8	9:99	0.43	6:18	0.52
9	9:13	0.40	1:123	0.48
10	9:74	0.39	17:22	0.43

* Adapted from Table 6 of Boddicker et al., 2014 (Ref. 4 in manuscript)

Supplementary Table S4. *cis*-eQTL SNPs that overlapped with the GWAS regions

VL GWAS region	cis-eQTL SNP	Chr	Position	Associated with VL?	cis-gene
Chr4:99MB	ASGA0020882	Chr4	99102754	Y	FCRL6
	H3GA0013452	Chr4	99503615	Y	FCER1A
	ASGA0020902	Chr4	99808905	Y	CD1D
Chr4:139MB	ALGA0029510	Chr4	139460507	Y	GBP5, GBP6
	INRA0017729	Chr4	139501558	Y	GBP5
	ASGA0023322	Chr4	139599066	Y	GBP5
	MARC0056249	Chr4	139642933	Y	GBP5
	WUR10000125	Chr4	139666819	Y	GBP5
	ALGA0029524	Chr4	139694383	Y	GBP5
	ASGA0023344	Chr4	139772782	Y	GBP5
	MARC0014819	Chr4	139800533	Y	GBP5
Chr7:130MB	MARC0049053	Chr7	13091459	N	GMPR

WG GWAS region	cis-eQTL SNP	Chr	Position	Associated with WG?	cis-gene
Chr1:123MB	MARC0087646	Chr1	123415670	Y	ICE2
	MARC0033480	Chr1	123441355	Y	ICE2
	INRA0003823	Chr1	123593103	N	ICE2
Chr3:138MB	ALGA0115665	Chr3	138589119	N	CMPK2
	MARC0089811	Chr3	138684370	Y	CMPK2
Chr4:139MB	ALGA0029510	Chr4	139460507	N	GBP5
	INRA0017729	Chr4	139501558	Y	GBP5
	ASGA0023322	Chr4	139599066	Y	GBP5
	MARC0056249	Chr4	139642933	Y	GBP5
	WUR10000125	Chr4	139666819	Y	GBP5
	ALGA0029524	Chr4	139694383	Y	GBP5
	ASGA0023344	Chr4	139772782	Y	GBP5
	MARC0014819	Chr4	139800533	Y	GBP5
Chr5:72MB	ALGA0032762	Chr5	72553804	N	USP18
Chr7:27MB	H3GA0020408	Chr7	27082547	N	CSNK2B
	H3GA0020425	Chr7	27300895	Y	CCHCR1
	M1GA0009773	Chr7	27470374	Y	PPT2
	UMB10000108	Chr7	27724510	N	SLA-7
	M1GA0009779	Chr7	27778363	N	GPANK1
	ASGA0032033	Chr7	27797198	N	GPANK1
	H3GA0020450	Chr7	27855827	N	CCHCR1
	DIAS0000302	Chr7	27986296	N	CCHCR1
Chr17:22MB	ASGA0075707	Chr17	22547070	N	MKKS

The cis-eQTL SNPs found associated with the VL/WG phenotypes are shaded grey.

Supplementary Table S5. Details of allele-specific expression test results

DPI	*cis*-eQTL SNP	*cis*-gene	Genotype at the *cis*-eQTL SNP (Ref:Alt)	Nr. individuals heterozygous at the *cis*-eQTL SNP	Total reads overlapping Ref/Alt allele at the heterozygous exonic SNPs of the *cis*-gene in phase with the *cis*-eQTL SNP		Binomial ratio corrected for overdispersion (Ref:Alt)	Beta Binomial likelihood ratio test p-value
					Ref	Alt		
0	WUR10000125	GBP5	[A:G]	11	239	685	0.29	3.17E-07
	ASGA0023322	GBP5	[C:T]	14	264	693	0.32	1.26E-06
	ASGA0023344	GBP5	[C:T]	11	270	670	0.31	1.44E-06
	INRA0017729	GBP5	[T:C]	11	296	655	0.32	8.69E-06
	ALGA0029524	GBP5	[T:G]	14	271	663	0.32	1.17E-05
	MARC0056249	GBP5	[T:C]	11	403	553	0.33	3.19E-05
	ALGA0029510	GBP5	[A:G]	15	311	565	0.35	0.001902755
	H3GA0020425	CCHCR1	[C:T]	12	277	472	0.42	0.028916636
	ALGA0029510	GBP6	[A:G]	15	237	311	0.47	0.242189150
	MARC0089811	CMPK2	[A:G]	10	835	803	0.51	0.673555302
	ASGA0020902	CD1D	[T:G]	17	430	472	0.50	0.932508999
4	ASGA0023322	GBP5	[C:T]	17	422	1059	0.32	8.01E-12
	WUR10000125	GBP5	[A:G]	15	422	1059	0.32	3.01E-10
	INRA0017729	GBP5	[T:C]	15	422	1059	0.33	3.48E-10
	ASGA0023344	GBP5	[T:C]	15	422	1059	0.33	3.48E-10
	MARC0056249	GBP5	[T:C]	15	422	1059	0.33	3.90E-10
	ALGA0029524	GBP5	[T:G]	18	466	968	0.35	5.97E-08
	ALGA0029510	GBP6	[A:G]	18	535	1002	0.40	2.01E-06
	ALGA0029510	GBP5	[A:G]	18	498	907	0.38	2.05E-05
	MARC0089811	CMPK2	[A:G]	12	1581	1640	0.47	0.052111577
	ASGA0020902	CD1D	[T:G]	19	447	477	0.48	0.243556855
	H3GA0020425	CCHCR1	[T:C]	14	208	231	0.50	0.92239505
7	WUR10000125	GBP5	[A:G]	15	404	979	0.33	1.10E-08
	MARC0056249	GBP5	[T:C]	15	391	939	0.35	5.20E-08
	ALGA0029510	GBP5	[A:G]	18	383	930	0.36	5.17E-07
	INRA0017729	GBP5	[T:C]	15	542	808	0.36	1.28E-06
	ALGA0029510	GBP6	[A:G]	18	537	852	0.42	2.93E-05
	ALGA0029524	GBP5	[T:G]	19	428	855	0.38	7.95E-05
	ASGA0023344	GBP5	[T:C]	15	516	774	0.38	7.97E-05
	ASGA0023322	GBP5	[C:T]	17	556	688	0.40	8.52E-04
	H3GA0020425	CCHCR1	[C:T]	14	379	623	0.45	0.037304603
	ASGA0020902	CD1D	[T:G]	20	480	513	0.48	0.287435853
	MARC0089811	CMPK2	[A:G]	12	1274	1426	0.49	0.481593663

DPI	*cis*-eQTL SNP	*cis*-gene	Genotype at the *cis*-eQTL SNP (Ref:Alt)	Nr. individuals heterozygous at the *cis*-eQTL SNP	Total reads overlapping Ref/Alt allele at the heterozygous exonic SNPs of the *cis*-gene in phase with the *cis*-eQTL SNP		Binomial ratio corrected for overdispersion (Ref:Alt)	Beta Binomial likelihood ratio test p-value
					Ref	Alt		
11	WUR10000125	GBP5	[A:G]	14	383	1059	0.32	5.41E-09
	ALGA0029510	GBP6	[A:G]	16	417	825	0.37	1.04E-07
	ASGA0023344	GBP5	[T:C]	14	459	1048	0.36	1.96E-06
	INRA0017729	GBP5	[T:C]	14	582	1001	0.38	2.83E-05
	ASGA0023322	GBP5	[C:T]	16	505	974	0.38	3.24E-05
	ALGA0029510	GBP5	[A:G]	16	543	828	0.43	0.027946377
	MARC0056249	GBP5	[T:C]	14	588	897	0.43	0.034632517
	H3GA0020425	CCHCR1	[T:C]	12	292	563	0.44	0.050485365
	ALGA0029524	GBP5	[T:G]	18	887	508	0.56	0.106048889
	MARC0089811	CMPK2	[A:G]	9	627	650	0.49	0.709404063
	ASGA0020902	CD1D	[T:G]	18	281	298	0.51	0.713080159
14	ASGA0023322	GBP5	[C:T]	19	841	2056	0.31	1.22E-13
	WUR10000125	GBP5	[A:G]	16	889	2075	0.35	1.84E-08
	MARC0056249	GBP5	[T:C]	16	894	2094	0.35	6.67E-08
	INRA0017729	GBP5	[T:C]	16	902	2073	0.36	5.61E-07
	ALGA0029510	GBP5	[A:G]	20	880	2046	0.37	8.74E-07
	ALGA0029524	GBP5	[T:G]	20	891	2059	0.36	1.49E-06
	ALGA0029510	GBP6	[A:G]	20	902	1588	0.43	2.99E-06
	ASGA0023344	GBP5	[T:C]	16	1143	1851	0.40	2.53E-04
	H3GA0020425	CCHCR1	[C:T]	12	322	586	0.41	0.00464776
	ASGA0020902	CD1D	[T:G]	23	635	572	0.53	0.06096316
	MARC0089811	CMPK2	[G:A]	12	918	1030	0.50	0.997481794

Supplementary Table S6. Detailed statistics on eQTL and phenotype associations for the final candidate genes

SNP | Ensembl ID | Gene symbol	eQTL			viral load			weight gain		
	day	beta	q-val	day	beta	p-value	day	beta	p-value
**WUR10000125* | ENSSSCG00000024973 | *GBP5***	d00	3.18E-01	9.66E-02	[redacted]			d07	9.30E-02	2.05E-01
	d04	3.55E-01	3.32E-01	d04	-1.45E-01	6.63E-03	d14	4.21E-01	6.76E-04
	d07	3.56E-01	3.48E-02	d07	-9.76E-02	8.40E-04	d21	9.63E-01	4.59E-07
	d11	4.25E-01	3.68E-02	d11	-2.55E-01	1.70E-10	d28	1.19E+00	1.34E-06
	d14	4.91E-01	2.70E-02	d14	-2.85E-01	7.43E-07	d35	1.45E+00	3.65E-06
							d42	1.83E+00	8.82E-07
**ALGA0029510 | ENSSSCG00000030801| *GBP6***	d00	1.89E-01	2.85E-01	[redacted]					
	d04	1.00E-01	7.54E-01	d04	-1.01E-01	3.80E-02			
	d07	2.70E-01	2.49E-02	d07	-4.47E-02	9.40E-02			
	d11	2.44E-01	1.32E-01	d11	-1.44E-01	9.79E-05			
	d14	2.85E-01	8.12E-02	d14	-1.34E-01	1.16E-02			
**H3GA0020425 | ENSSSCG00000001391 | *CCHCR1***	d00	-1.38E-01	1.95E-01				d07	6.05E-02	3.53E-01
	d04	-1.05E-01	4.89E-01				d14	1.49E-01	1.80E-01
	d07	-1.74E-01	3.48E-02				d21	5.33E-01	1.89E-03
	d11	-1.13E-01	4.33E-01				d28	5.94E-01	7.29E-03
	d14	-1.74E-01	2.70E-02				d35	7.94E-01	4.76E-03
							d42	1.16E+00	4.72E-04
**MARC0089811 | ENSSSCG00000008647 | *CMPK2***	d00	6.26E-02	7.87E-01				d07	-8.50E-02	1.76E-01
	d04	1.00E-01	6.35E-01				d14	-1.91E-01	7.31E-02
	d07	2.07E-01	4.42E-02				d21	-3.87E-01	1.96E-02
	d11	6.49E-03	9.04E-01				d28	-5.44E-01	1.08E-02
	d14	9.59E-02	5.22E-01				d35	-7.63E-01	4.85E-03
							d42	-8.18E-01	1.10E-02

* There are multiple cis-eQTL SNPs in the chr4:139 Mb region of which the WUR SNP shown here is amongst the most significant eQTL and phenotype QTL.
